# sybil – Efficient constraint-based modelling in R

**DOI:** 10.1186/1752-0509-7-125

**Published:** 2013-11-13

**Authors:** Gabriel Gelius-Dietrich, Abdelmoneim Amer Desouki, Claus Jonathan Fritzemeier, Martin J Lercher

**Affiliations:** 1 Institute for Computer Science, Heinrich-Heine-University, Universitätsstr 1, 40225 Düsseldorf, Germany

**Keywords:** Constraint-based modelling, Flux-balance analysis, FBA, MOMA, ROOM, GNU R

## Abstract

**Background:**

Constraint-based analyses of metabolic networks are widely used to simulate the properties of genome-scale metabolic networks. Publicly available implementations tend to be slow, impeding large scale analyses such as the genome-wide computation of pairwise gene knock-outs, or the automated search for model improvements. Furthermore, available implementations cannot easily be extended or adapted by users.

**Results:**

Here, we present sybil, an open source software library for constraint-based analyses in R; R is a free, platform-independent environment for statistical computing and graphics that is widely used in bioinformatics. Among other functions, sybil currently provides efficient methods for flux-balance analysis (FBA), MOMA, and ROOM that are about ten times faster than previous implementations when calculating the effect of whole-genome single gene deletions *in silico* on a complete *E. coli* metabolic model.

**Conclusions:**

Due to the object-oriented architecture of sybil, users can easily build analysis pipelines in R or even implement their own constraint-based algorithms. Based on its highly efficient communication with different mathematical optimisation programs, sybil facilitates the exploration of high-dimensional optimisation problems on small time scales. Sybil and all its dependencies are open source. Sybil and its documentation are available for download from the comprehensive R archive network (CRAN).

## Background

Constraint-based analyses have become a widely used tool for the study of genome-scale biochemical reaction networks [1]. The most prominent of these methods is flux-balance analysis (FBA). Here, metabolite fluxes through biochemical reactions are constrained by the conservation of mass, by thermodynamics (reaction directionality), by the assumption of a steady state for internal metabolite concentrations, and by the availability of nutrients. These constraints are used as boundary conditions for a linear optimisation problem, in which a biologically motivated objective function — often the yield of biomass production — is maximised. The result is a distribution of metabolic fluxes across the network, comprising a metabolic phenotype (or functional state) of the network [2-4].

Apart from such constraint-based optimisation methods, several other tools that use different philosophies for metabolic modelling are available. One example is the computation of elementary flux modes to represent the feasible solution space of a metabolic network [5]. Another approach is structural kinetic modelling, i.e., the description of dynamical properties of metabolic networks in combination with experimental data [6].

Several tools for constraint-based optimisation analyses are currently available (reviewed in [7-9]). The most widely used software is the COBRA Toolbox [10] for MATLAB. While these tools provide implementations of FBA and other constraint-based methods, they are relatively slow when applied to large series of simulations (e. g., when calculating the biomass yield of all double-gene knockouts in a unicellular organism). Further, available implementations mostly require licenses for MATLAB, and are not flexible enough to allow users to easily design their own large-scale analyses. For metabolic networks for which elementary modes [11] or extreme pathways [12,13] can be calculated, such higher-level descriptions of the solution space may provide fast alternatives to the constrained-based algorithms implemented in sybil.

The R computer language has become the standard programming environment in many scientific fields that depend on numerical data analysis, in particular in the analysis of biological high-throughput data. However, R currently offers only very limited options for constraint-based analyses. The R package BiGGR [14] provides access to the BiGG database [15] and can perform flux-balance analysis, visualising the results as graphs. The R package abcdeFBA [16] provides flux-balance analysis and phenotypic phase plane analysis. However, both packages are limited in scope and lack flexibility.

With sybil, which shares some functionality with the COBRA Toolbox and the R packages described above, we aim to establish R as a major platform for constraint-based analyses of biological networks. Besides offering powerful analysis tools in a versatile and freely available environment, sybil aims to supersede previous implementations in terms of calculation speed, flexibility and extensibility.

## Implementation

Sybil is implemented in the R programming language [17] as an object oriented library (Additional file 1). The design of some of its functions was inspired by the COBRA Toolbox [10]. Once the sybil library is loaded into the R environment, the user can access a range of functions to read and manipulate metabolic network models, to perform different constraint-based calculations, and to visualise the results.

Sybil is programmed for both speed and memory efficiency; in our experience, about 1 GB of RAM should be sufficient for all types of analyses, even when performed on the largest complete single cell type metabolic models currently available.

Sybil provides a set of "high-level" functions to access frequently used complex algorithms with a single function call (e. g., fluxVar() for flux variability calculations [18,19], or geneDeletion() for prediction of gene deletion effects). Another way to use sybil is to directly use "low-level" functions (e. g., optimizeProb() or any of the API-functions from the linked optimiser software). Methods implemented in class sysBiolAlg provide a particularly comfortable way to execute constraint-based analyses involving optimisation steps (FBA and related algorithms): here, the class takes care of the optimisation software without user interference. Sybil’s architecture provides the user with a highly flexible and adjustable framework. Sybil is equally suited for off-the-shelf constraint-based analyses, for building complex analysis pipelines, and for the development of new constraint-based analysis methods.

**Figure 1:**
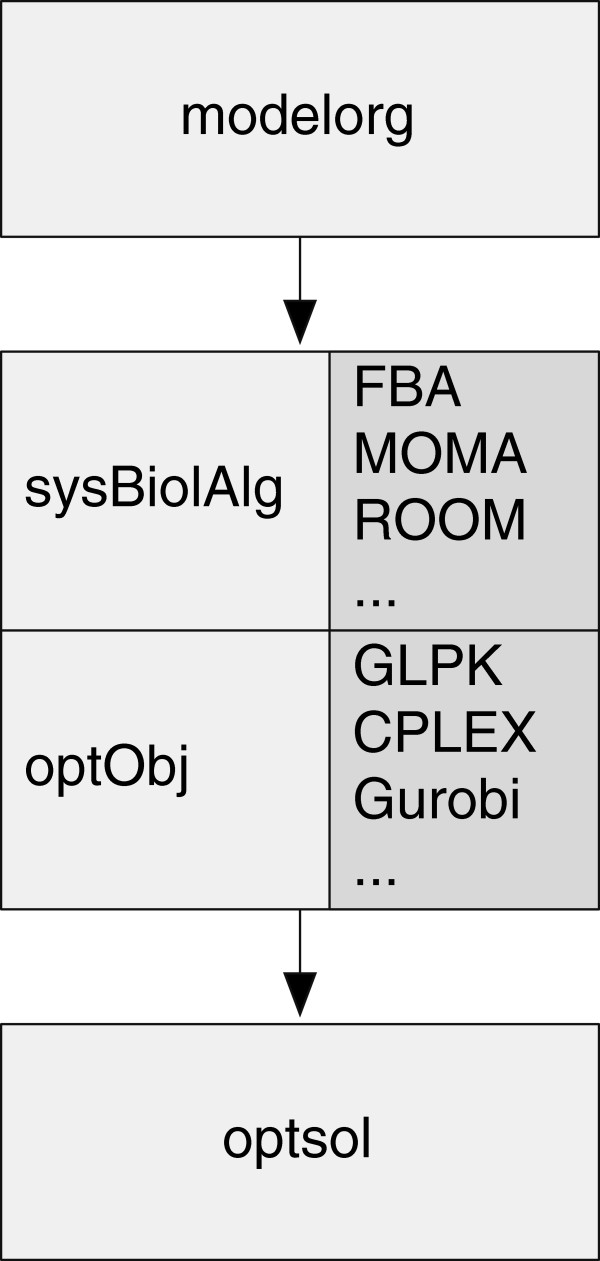
**Main classes in sybil.** Class modelorg serves as sybil’s representation of a metabolic model. Instances of that class harbour the stoichiometric matrix, reaction names and properties, metabolites names, and gene to reaction associations. An instance of class optObj is basically a pointer to the problem object generated by the mathematical programming software. Class optObj is used for communication to the solver software. It provides methods to create, modify and solve optimisation problems, independent of the used solver software. Class sysBiolAlg holds concrete instances of class optObj which are prepared for a specific model to use with a particular algorithm (e. g. FBA or MOMA). Class sysBiolAlg is the entry point for new algorithms in sybil. The default constructor generates problem objects. Classes extending class sysBiolAlg only need to describe a specific algorithm as a formal optimisation problem. Knowledge about the details of the solver software is not required. Class optsol harbors the results of an optimisation analysis and contains analysis-specific plotting methods.

The implementation of sybil follows the object oriented programming paradigm. Figure 1 shows the connection between the important classes implemented in sybil. Class modelorg contains sybil’s representation of a metabolic network.

A number of functions are available to manipulate metabolic network models, such as addReact() to add new reactions to the model, changeGPR() to alter the gene-reaction association rules, and changeUptake() and editEnvir() to change the modelled environments. Instances of class sysBiolAlg contain a pointer to the problem object, comprised of metabolic model, constraints, and analysis algorithm to be used. For applications that involve repetitive analyses, such as flux variability or genome-wide knockout studies, the problem object used by the optimisation software is prepared only once as an instance of class sysBiolAlg. Modifications to the problem required in the course of the analysis are then applied at the level of class sysBiolAlg, so that the problem object must not be re-created for every optimisation. The results returned by the mathematical programming software are stored in instances of class optsol.

## Results and discussion

### Key features

Sybil provides several functions to perform constraint-based analyses of metabolic networks. Genetic perturbations can be simulated through FBA [2,3], minimisation of metabolic adjustment (MOMA) [20], a linear version of the MOMA algorithm similar to [21], or regulatory on/off minimisation (ROOM) [22]. Additionally, sybil can perform flux variability (FVA) [18,19], robustness [23], and phenotypic phase plane (PhPP) [24,25] analyses (see Additional file 2 for a comparison with other constraint-based analysis tools). The implementations are optimised for speed when running a large number of similar optimisations on the same model (e. g. genome-wide gene deletion simulations).

Due to sybil’s object oriented implementation, users can easily add new functions. Class sysBiolAlg can be extended to implement additional algorithms, which are then available to high-level functions in sybil without further user interaction. Like other toolboxes for constraint-based analyses, sybil communicates with external mathematical optimisation software (e. g., GLPK) to generate and solve various types of optimisation problems. This process is handled by class optObj, which provides a large set of methods to generate, modify, and solve mathematical programming problems and to access the results; the user does not need any deeper knowledge about the differences of the various solvers that can be used by sybil. However, if necessary, all parameters available within the solver software can be accessed directly in sybil.

In the future, we plan to further extend sybil, e. g. by adding methods that incorporate gene expression data into an FBA approach [26-29]. Two such addition are already implemented in the separate R packages sybilDynFBA [30], which uses dynamic FBA simulations to predict concentration changes of external metabolites as described in [31], and sybilEFBA [32] using gene expression data to improve FBA predictions. Another available add-on to sybil is the R package RSeed [33], which analyses network topology to identify metabolites that must be acquired from the environment [34]. The R package sybilSBML (Additional file 3) adds SBML support to sybil.

### Calculation speed

The calculation speed of the optimisations depends on the mathematical optimisation software used. Typically, for large mathematical problems, IBM ILOG CPLEX is slightly faster than the two freely available solvers GLPK and COIN-OR Clp (see below). However, major differences in the running times of different constraint-based analysis tools stem mostly from the overheads produced by the communication between the main program and the solver. This overhead is minimised by sybil through purpose-built fast interfaces to the C-API of each package.

Most of the implemented algorithms require the generation of an optimisation problem based on the model, the constraints, and the desired algorithm (such as FBA or linear MOMA). During batch calculations, only small changes to the optimisation problem are required, e. g., changes of variable bounds in an *in silico* gene deletion experiment, or alteration of the objective function during flux variability analysis. To speed up iterations over many such small changes, the optimisation problem is formulated only once; all changes are then applied directly to the pre-formed optimisation problem of the mathematical optimisation software.

**Figure 2:**
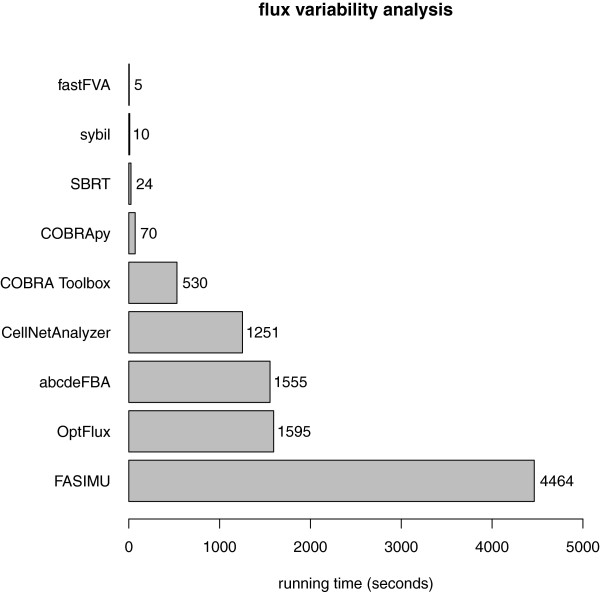
**Running times for flux variability analysis.** Running time of flux variability analysis in various software packages using the GNU Linear Programming Kit (GLPK). Time was measured in R with the function Rprof() and in MATLAB with the Profiler function. In both cases, the running time of a function is reported as "total time". For OptFlux, the Unix command top in delta mode was used. SBRT itself reports the elapsed time for flux variability analysis. For FASIMU, the "real time" reported by the Unix command time was used. For COBRApy, the Python module time was used. All simulations were run ten times on the same desktop PC (Intel Xeon, 2.8 GHz, running Mac OS 10.7). The reported running times are arithmetic means of these values.

**Figure 3:**
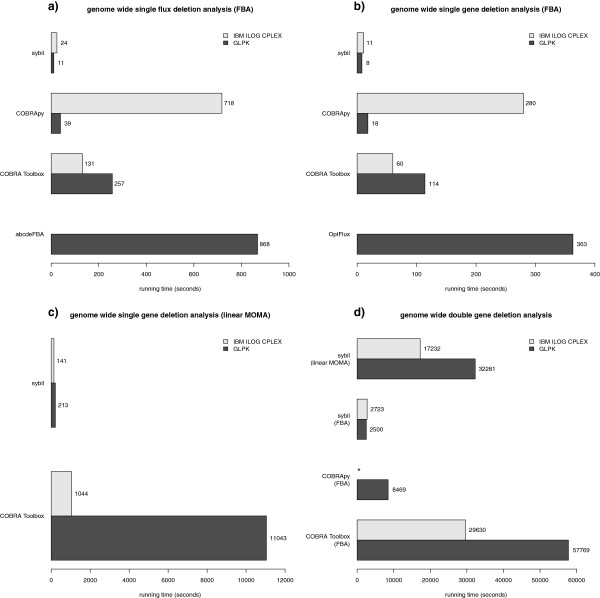
**Running time for simulations of genome-wide genetic perturbations.** Running time of genome-wide *in silico* perturbation experiments (single- and double-gene knockouts) using various software packages and different mathematical optimisation packages. **a)** For computation of single flux deletions, we used the functions oneFluxDel() in sybil, single_deletion() in COBRApy, singleRxnDeletion() in the COBRA Toolbox, and Exhaustive_single_deletion() in abcdeFBA. **b-d)** In sybil, we used the functions oneGeneDel() and doubleGeneDel() for simulations of single and pairwise knockout mutants, respectively. The same results were achieved with the COBRA Toolbox using functions singleGeneDeletion() and doubleGeneDeletion(), respectively. In COBRApy, we used functions single_deletion() and double_deletion. In OptFlux, the calculation of so called "critical genes" was used. The running times for COBRApy are extremely long when using the IBM ILOG CPLEX solver (* more than 24 hours for a simulation of all pairwise knockout mutants). The interface between COBRApy and IBM ILOG CPLEX seems not to support reusing a computed basis for re-solving an optimisation problem. Running times were obtained as in Figure 2.

Figures 2 and 3 compare the running times of different implementations of typical algorithms used in constraint-based modelling; they also illustrate the impact of different mathematical optimisation programs on calculation speed. For all calculations, we used a complete model of *E. coli* metabolism, containing 2382 reactions and 1261 independent genes [35].

Figure 2 shows the performance of different implementations of genome-wide flux variability analysis (FVA) [18,19] using GLPK as the mathematical optimisation program. For FVA, an optimal growth rate was estimated by FBA. Then, for all reactions in the model, we computed the minimal and maximal flux at this growth rate. The software tools fastFVA [36], COBRA Toolbox [10], and CellNetAnalyzer [37] implement FVA for the MATLAB environment; SBRT [38] and OptFlux [39] are Java-based implementations; FASIMU [40] is implemented in bash and awk; COBRApy [41] is a Python implementation; and abcdeFBA and sybil provide R implementations. All these software packages perform FVA with a single function call.

As can be seen in Figure 2, fastFVA is the fastest implementation of the flux variability algorithm. The main algorithm is fully implemented in C++ and can be accessed from within MATLAB as an extension to the COBRA Toolbox. The C++ implementation results in a very fast running time, but makes the program inflexible; only flux variability analysis can be performed, and changes to the solver software parameters require modification of the source code. Sybil, the second fastest implementation, is — compared to other implementations — only slightly slower than fastFVA. Sybil’s optimisations make use of wrapper functions (in this case through the R package glpkAPI [42]), allowing access to the C-API of the mathematical programming software from within R. This combines very short running times with flexible communication with the solver software. SBRT uses its own Java interface to GLPK (and IBM ILOG CPLEX), which is in function similar to the wrapper software used by sybil. In COBRApy, a separate Python module provides a connection to GLPK. The MATLAB packages COBRA Toolbox and CellNetAnalyzer make use of glpkmex [43], which provides high-level function calls to build and solve mathematical programming problems in one step. This architecture results in longer running times, as the problem needs to be rebuilt for every step in flux variability analysis, even if only minor adjustments to the model are required. The R package abcdeFBA uses the R package Rglpk [44], which works similar to glpkmex. OptFlux and FASIMU use the command line interfaces of GLPK and IBM ILOG CPLEX (FASIMU) or COIN-OR Clp (OptFlux) and generate the necessary input files for every optimisation. FASIMU computes the optimisations one by one, resulting in the longest running time, while OptFlux can run — to some extent — optimisations simultaneously.

Figure 3 shows the performance of genome-wide *in silico* gene deletion experiments with the same complete model of *E. coli* metabolism used for the flux variability analyses. Regardless of the details of the experiment (gene vs. flux deletions; single- vs. double-gene deletions; FBA vs. linear MOMA), sybil clearly outperforms other implementations in terms of computation speed; this is achieved through the efficient handling of optimisation problems that repeatedly need to be re-optimised, but do not change very much from one optimisation to the other. Sybil was successfully used as the constraint-based core of a machine learning method to reconcile model predictions with genome-scale experimental double-gene knockout data [45]. In this study, we demonstrated the feasibility of automated metabolic model refinement by correcting misannotations in NAD biosynthesis in the metabolic model of yeast (iMM904, [46]).

Another fast tool is F2C2 [47], a MATLAB tool for flux coupling analysis which computes all blocked and coupled reactions of the *E. coli* model in less than five minutes on our test system.

### Examples

#### Reading model files

Sybil can read text-based representations of metabolic networks written in the 'Systems Biology Markup Language’ (SBML) [48], which is an extension of XML. A large range of models in this de-facto standard format is available from the web pages of the Palsson group at UCSD [49]. Each of these models is the outcome of an elaborate model-building process, which starts from database and literature searches and culminates in an iterative comparison of computational predictions and lab experiments. Details on how to reconstruct whole genome metabolic network models suitable for constraint based analyses are reviewed in [50,51]. A reconstruction of the central energy metabolism of *E. coli*[23] is included as an example dataset (Additional file 4). In order to read SBML files, the package sybilSBML (Additional file 3) from CRAN, which is itself powered by LibSBML [52], is required.

Sybil can also read models written in a column-based format, such as exported reaction lists of the BiGG database [15]. Example files for the central energy metabolism of *E. coli* are also included in this format (Additional file 4). These can be read in using the command readTSVmod() (assuming Additional file 4 is unpacked in the working directory of R):

The variable Ec_core now contains an *in silico* representation of the central energy metabolism of *E. coli* which can be used for further analysis. The definition of the column-based format is described in the sybil package vignette (Additional file 5).

#### Constraint-based analysis of metabolic networks

Genetic perturbations of metabolic networks can be simulated using the function geneDeletion(). The command

performs a single gene deletion analysis on the example dataset, using flux-balance analysis to determine reductions in metabolite production, and employing the mathematical optimisation software GLPK for the optimisations. The parameter ’combinations’ indicates the number of genes to knockout simultaneously in each optimisation. Setting this parameter to 2 results in the simulation of all possible pairwise gene knockouts, setting it to 3 will compute all triple-gene knockouts. Due to sybil’s streamlined communication with the solver software, which only transmits changes to the model rather than the full model for each deletion, this function helps to deal with the combinatorial explosion inherent in systematic multiple-gene knockout experiments. The parameter ’algorithm’ indicates the algorithm used to determine the functional state of the metabolic network after gene deletion. It can be set to 

• "fba": for flux-balance analysis (this is the default value) as described in [2,3],

• "mtf": for flux-balance analysis and additionally selecting the flux distribution resulting in the smallest absolute total flux,

• "moma": for minimisation of metabolic adjustment as described in [20],

• "lmoma": for a linear version of the MOMA algorithm similar to the version described in [21], or

• "room": for regulatory on/off minimisation as described in [22].

The parameter 'solver’ selects the mathematical optimisation software used by the algorithms. It can be set to 

• "glpkAPI": for GLPK [53], via R package glpkAPI [42],

• "cplexAPI": for IBM ILOG CPLEX [54], via R package cplexAPI [55],

• "clpAPI": for COIN-OR Clp [56], via R package clpAPI [57],

• "lpSolveAPI": for lp_solve [58], via R package lpSolveAPI [59], or

• "sybilGUROBI": for Gurobi [60], via R package sybilGUROBI.

All R packages are available on CRAN [61], with the exception of sybilGUROBI, which is available on request. The sybil package vignette (Additional file 5) contains further examples of constraint-based metabolic network analyses, such as flux variability or robustness analyses, as well as graphical representation of results and instructions for the interaction with mathematical optimisation programs.

## Conclusions

Sybil is designed to address large scale questions in reasonable time frames, making it possible to generate and run *in silico* experiments that result in high-dimensional optimisation problems. New algorithms can be easily implemented using the sybil framework and can be distributed as add-on packages to the systems biology community.

## Availability and requirements

**Project name:** sybil

**Project home page:**http://CRAN.R-project.org/package=sybil

**Operating system(s):** Platform independent

**Programming language:** R

**Other requirements:** A mathematical optimisation software (one of GLPK, IBM ILOG CPLEX, COIN-OR Clp, or lpSolve)

**License:** GNU GPL

## Abbreviations

FBA: Flux-balance analysis; FVA: Flux variability analysis; MOMA: Minimisation of metabolic adjustment; ROOM: Regulatory on/off minimisation; SBML: Systems biology markup language.

## Competing interests

The authors declare that they have no competing interests.

## Authors’ contributions

GGD developed the R packages sybil, sybilSBML, glpkAPI, clpAPI and cplexAPI, implemented the algorithms, conceived the handling of the mathematical programming software, and wrote the manuscript. AAD developed sybil add-on packages sybilDynFBA and sybilEFBA and tested and applied sybil and made suggestions for improvements and additions. CJF developed sybil add-on package RSeed and tested and applied sybil and made suggestions for improvements and additions. MJL initated the project, suggested improvements and additions to sybil, and contributed to the writing of the manuscript. All authors read and approved the final manuscript.

## Supplementary Material

Additional file 1 **R package sybil.** Archive of the sybil version current at submission. Click here for file

Additional file 2 Features contained in sybil and other commonly used software packages. Click here for file

Additional file 3 **R package sybilSBML.** Archive of the sybilSBML version current at submission. Click here for file

Additional file 4 **Example dataset.** Archive containing the input files required by sybil for the reconstruction of the central energy metabolism of *E. coli* in column based format and in SBML format. The files packaged into the archive can be read with the sybil command readTSVmod. Click here for file

Additional file 5 **sybil package vignette.** A user guide for sybil in PDF format. It can be accessed from within a running R session with the command vignette("sybil"). Click here for file
