# Supplementary material for: sybil – Efficient constraint-based modelling in R
Source: BMC Syst Biol. 2013 Nov 13;7:125. doi: 10.1186/1752-0509-7-125 (PMC3843580; doi:10.1186/1752-0509-7-125)
Supplement: Additional file 2 — Features contained in sybil and other commonly used software packages. [file 1752-0509-7-125-S2.pdf]

	Knockout mutants					Other algorithms									Input files		Network tools			
	FBA	gene knockouts (FBA)	MOMA	ROOM	series of knockouts[a]	FVA	total flux minimisation[b]	robustness analysis	PhPP[c]	dynamic FBA	series of optimisations[d]	flux coupling	sampling	elementary modes/ extreme pathways	SBML	column based[e]	fluxomics data	integration of gene expression data	network visualisation	network reconstruction
sybil	✓	✓	✓	✓	✓	✓	✓	✓	✓	✓[f]	✓		✓		✓[g]	✓		✓[h]		
abcdeFBA	✓					✓		✓												
BiGGR	✓																			
COBRA Toolbox	✓	✓	✓		✓	✓	✓	✓	✓	✓		✓	✓	✓	✓	✓	✓	✓	✓	✓
COBRApy	✓	✓	✓		✓	✓					✓				✓					
fastFVA						✓					✓									
CellNetAnalyzer	✓	✓				✓								✓	✓		✓		✓	✓
SBRT	✓	✓				✓					✓				✓					
OptFlux	✓	✓	✓	✓		✓									✓	✓				
FASIMU	✓	✓	✓			✓	✓								✓			✓	✓[i]	✓
F2C2												✓								

[a] In an automated fashion for a given set of genes, i. e., knocking out one gene per optimisation.
[b] Sum of absolute flux values.
[c] Phenotypic phase planes
[d] Optimised for speed by only applying changes rather than rebuilding the problem object.
[e] e. g., compatible to the output of the BiGG database.
[f] Add-on package sybilDynFBA required, available separately from CRAN.
[g] Add-on package sybilSBML required (Additional file 3).
[h] Add-on package sybilEFBA required, available separately from CRAN.
[i] Cytoscape required.
