# Supplementary material for: sybil – Efficient constraint-based modelling in R
Source: BMC Syst Biol. 2013 Nov 13;7:125. doi: 10.1186/1752-0509-7-125 (PMC3843580; doi:10.1186/1752-0509-7-125)
Supplement: Additional file 5 — sybil package vignette. A user guide for sybil in PDF format. It can be accessed from within a running R session with the command vignette("sybil"). [file 1752-0509-7-125-S5.pdf]

sybil – Efficient Constrained Based Modelling in R

Gabriel Gelius-Dietrich

September 8, 2013

Contents

1 Introduction **2**

2 Installation **2**

3 Input files **2**

3.1 Tabular form . . . 3

3.1.1 Field and entry delimiter . . . 3

3.1.2 Model description . . . 3

3.1.3 Metabolite list . . . 4

3.1.4 Reaction list . . . 4

3.1.5 How to write a reaction equation string . . . 5

3.2 SBML . . . 8

4 Usage **8**

4.1 Documentation . . . 8

4.2 Reading a model in tabular form . . . 9

4.3 Media conditions . . . 10

4.4 Flux-balance analysis . . . 12

4.5 Minimize total flux . . . 14

4.6 Genetic perturbations . . . 16

4.6.1 Minimization of metabolic adjustment (MOMA) . . . 16

4.6.2 Regulatory on/off minimization (ROOM) . . . 17

4.6.3 Multiple knock-outs . . . 17

4.7 Flux variability analysis . . . 21

4.8 Robustness analysis . . . 21

4.9 Phenotypic phase plane analysis . . . 22

4.10 Summarizing simulation results . . . 24

4.11 Parallel computing . . . 25

4.12 Interacting with the optimization process . . . 26

4.13 Optimization software . . . 27
4.14 Setting parameters to the optimization software . . . 28
4.14.1 GLPK . . . 28
4.14.2 IBM ILOG CPLEX . . . 28
4.14.3 COIN-OR Clp . . . 28
4.14.4 lpSolveAPI . . . 29
4.15 Setting parameters in sybil . . . 29
4.15.1 Solver software specific . . . 30
4.15.2 Analysis specific . . . 30

5 Central data structures **31**
5.1 Class modelorg . . . 31
5.2 Class optsol . . . 33
5.3 Class optObj . . . 35
5.4 Class sysBiolAlg . . . 38
5.4.1 Constructor methods . . . 39
5.4.2 New algorithms . . . 40

1 Introduction

The R-package *sybil* is a Systems Biology Library for R, implementing algorithms for constraint based analysis of metabolic networks. Among other functions, *sybil* currently provides efficient methods for flux-balance analysis (FBA), minimization of metabolic adjustment (MOMA), regulatory on/off minimization (ROOM), flux variability Analysis and robustness Analysis. The package *sybil* makes use of the sparse matrix implementation in the R-package *Matrix*.

2 Installation

The package *sybil* itself depends on an existing installation of the package *Matrix*. In order to run optimizations, at least one of the following additional R-packages and the corresponding libraries are required: *glpkAPI*, *cplexAPI*, *clpAPI* or *lpSolveAPI*. These packages are available from CRAN[1]. Additionally, *sybilGUROBI*—supporting the Gurobi optimizer[2]—is available on request.

3 Input files

Input files for *sybil* are text files containing a description of the metabolic model to analyze. These descriptions are basically lists of reactions. Two fundamentally different types of text files are supported: i) in tabular form (section 3.1), or ii) in SBML format (section 3.2).

[1] `http://cran.r-project.org/`
[2] `http://www.gurobi.com`

Table 1: Field and entry delimiters and their default values.

variable	default value
`fielddelim`	`\t`
`entrydelim`	`,␣`

3.1 Tabular form

Models in tabular form can be read using the function `readTSVmod()` and written using the function `modelorg2tsv()`. Each metabolic model description consists of three tables:

1. A model description, containing a model name, the compartments of the model and so on (section 3.1.2).
2. A list of all metabolites (section 3.1.3).
3. A list of all reactions (section 3.1.4).

A model must contain at least a list of all reactions. All other tables are optional. The tables contain columns storing the required data. Some of these columns are optional, but if a certain table exists, there must be a minimal set of columns. The column names (the first line in each file) are used as keywords and cannot be changed.

3.1.1 Field and entry delimiter

There are two important variables in connection with text based tables: The fields (columns) of the tables are separated by the value stored in the variable `fielddelim`. If a single entry of a field contains a list of entries, they are separated by the value of the variable `entrydelim`. The default values are given table 1. The default behavior is, that the columns of each table are separated by a single `tab` character. If a column entry holds more than one entry, they are separated by a comma followed by a single whitespace ␣ (not a tab!).

3.1.2 Model description

Every column in this table can have at most one entry, meaning each entry will be a single character string. If a model description file is used, there should be at least the two columns `name` and `id`. If they are missing—or if no model description file is used—they will be set to the file name of the reaction list, which must be there (any file name extension and the string `_react` at the end of the file name, will be removed). The model description file contains the following fields:

name A single character string giving the model name. If this field is empty, the filename of the reaction list is used.

id A single character string giving the model id. If this field is empty, the filename of the reaction list is used.

description A single character string giving a model description (optional).

compartment A single character string containing the compartment names. The names must be separated by the value of `fielddelim` (optional, see section 3.1.1).

abbreviation A single character string containing the compartment abbreviations. The abbreviations must be in square brackets and separated by the value of `fielddelim` as mentioned above (optional).

Nmetabolites A single integer value giving the number of metabolites in the model (optional).

Nreactions A single integer value giving the number of reactions in the model (optional).

Ngenes A single integer value giving the number of unique, independent genes in the model (optional).

Nnnz A single integer value giving the number of non-zero elements in the stoichiometric matrix of the model (optional).

The file `Ec_core_desc.tsv` (in `extdata/`) contains an exemplarily table for the core energy metabolism of *E. coli* [Palsson, 2006, Orth et al., 2010a].

3.1.3 Metabolite list

This table is used in order to match metabolite id's given in the list of reactions to long metabolite names. This table is optional, but if it is used, the columns `abbreviation` and `name` should not be empty.

abbreviation A list of single character strings containing the metabolite abbreviations.

name A list of single character strings containing the metabolite names.

compartment A list of character strings containing the metabolite compartment names. Each entry can contain more than one compartment name, separated by `fielddelim` (optional, currently unused).

The file `Ec_core_met.tsv` (in `extdata/`) contains an exemplarily table for the core energy metabolism of *E. coli* [Palsson, 2006, Orth et al., 2010a].

3.1.4 Reaction list

This table contains the reaction equations used in the metabolic network.

abbreviation A list of single character strings containing the reaction abbreviations (optional, if empty, a warning will be produced). Entries in the field abbreviation are used as reaction id's, so they must be unique. If they are missing, they will be set to $v_i,\ i \in \{1, \ldots, n\}\ \forall i$ with n being the total number of reactions.

name A list of single character strings containing the reaction names (optional, if empty, the reaction id's (abbreviations) are used as reaction names.

equation A list of single character strings containing the reaction equation. See section 3.1.5 for a description of reaction equation strings.

reversible A list of single character strings indicating if a particular reaction is reversible or not. If the entry is set to `Reversible` or `TRUE`, the reaction is considered as reversible, otherwise not. If this column is not used, the arrow symbol of the reaction string is used (optional, see section 3.1.5).

compartment A list of character strings containing the compartment names in which the current reaction takes place. Each entry can contain more than one name, separated by `fielddelim` (optional, currently unused).

lowbnd A list of numeric values containing the lower bounds of the reaction rates. If not set, zero is used for an irreversible reaction and the value of `def_bnd * -1` for a reversible reaction. See documentation of the function `readTSVmod` for the argument `def_bnd` (optional).

uppbnd A list of numeric values containing the upper bounds of the reaction rates. If not set, the value of `def_bnd` is used. See documentation of the function `readTSVmod` for the argument `def_bnd` (optional).

obj_coef A list of numeric values containing objective values for each reaction (optional, if missing, zero is used).

rule A list of single character strings containing the gene to reaction associations (optional).

subsystem A list of character strings containing the reaction subsystems. Each reaction can belong to more than one subsystem. The entries are separated by `fielddelim` (optional).

The file `Ec_core_react.tsv` (in `extdata/`) contains an exemplarily table for the core energy metabolism of *E. coli* [Palsson, 2006, Orth et al., 2010a].

3.1.5 How to write a reaction equation string

Any reaction string can be written without space. They are not required but showed here, in order to make the string more human readable.

Compartment Flag Each reaction string may start with a compartment flag in square brackets followed by a colon. The compartment flag here gives the location of all metabolites appearing in the reaction.

```
[c] :
```

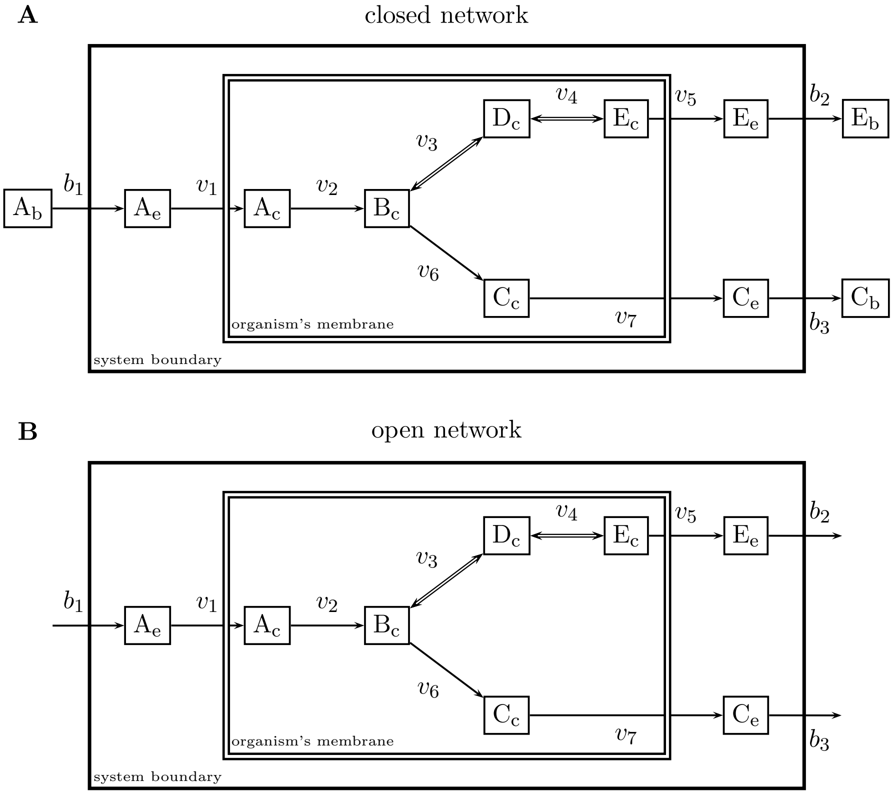

Figure 1: Simple example network. A) showing a closed network, B) an open network. Capital letters are used as metabolite id's, lower case letters are used as compartment id's: b: boundary metabolite, c: cytosol and e: external metabolite. Internal reactions are named $v_{1:7}$, transport reactions $b_{1:3}$. Reactions v_3 and v_4 are reversible, all others are irreversible. Metabolites $\mathrm{A_b}$, $\mathrm{C_b}$ and $\mathrm{E_b}$ are boundary metabolites and will be removed in order to obtain an open network.

The compartment flag can consist of more than one letter and—if used—must be an element of the field `abbreviation` in the model description. The letter `b` is reserved for boundary metabolites (argument `extMetFlag` in function `readTSVmod()`), which can be transported inside the system (those metabolites are only used in closed systems and will be removed during file parsing).

If the reaction string does not start with a compartment flag, the flag may be appended (without whitespace) to each metabolite id (e. g. for transport reactions):

```
h2o[e] <==> h2o[c]
```

If no compartment flag is found, it is set to `[unknown]`.

Reaction Arrow All reactions must be written in the direction educt to product, so that all metabolites left of the reaction arrow are considered as educts, all metabolites on the right of the reaction arrow are products.

The reaction arrow itself consists of one or more `=` or `-` symbols. The last symbol must be a `>`. If a reaction arrow starts with `<`, it is taken as reversible, if the field `reversible` in the reaction list is empty. If the field `reversible` is set to `TRUE` or `Reversible`, the reactions will be treated as a reversible reaction, independent of the reaction arrow. Each reaction must contain exactly one reaction arrow.

Stoichiometric Coefficients Stoichiometric coefficients must be in round brackets in front of the corresponding metabolite:

```
(2) h[c] + (0.5) o2[c] + q8h2[c] --> h2o[c] + (2) h[e] + q8[c]
```

Setting the stoichiometric coefficient in brackets makes it possible for the metabolite id to start with a number.

Examples A minimal reaction list without compartment flags for figure 1B (open network):

```
equation
A  --> B
B <==> D
D <==> E
B  --> C
   --> A
C  -->
E  -->
```

The same as above including compartment flags and external metabolites and all transport reactions for figure 1A (closed network). The reactions which take place in only one compartment (do not include a transport of metabolites across membranes) have their compartment flag at the beginning of the line (`[c]` in this example). For transport reactions all metabolites have their own compartment flag, e. g. in line 5 metabolite `A` is transported from compartment `[e]` (external) to compartment `[c]` (cytosol):

```
equation
[c]: A  --> B
[c]: B <==> D
[c]: D <==> E
[c]: B  --> C
A[e] --> A[c]
C[c] --> C[e]
E[c] --> E[e]
A[b] --> A[e]
C[e] --> C[b]
E[e] --> E[b]
```

The same as above including reaction id's for figure 1 (fields are separated by tabulators):

```
abbreviation    equation
v2    [c]: A  --> B
v3    [c]: B <==> D
v4    [c]: D <==> E
v6    [c]: B  --> C
v1    A[e] --> A[c]
v7    C[c] --> C[e]
v5    E[c] --> E[e]
b1    A[b] --> A[e]
b3    C[e] --> C[b]
b2    E[e] --> E[b]
```

3.2 SBML

In order to read model files written in systems biology markup language (SBML), the package *sybilSBML* is required, which is available from CRAN[3].

4 Usage

In the following sections, it is assumed, that package *glpkAPI* is installed additionally to *sybil*, thus GLPK is used as optimization software. Load *sybil* in a running R session:

```
> library(sybil)
```

4.1 Documentation

Get a list of all functions provided with *sybil*:

```
> library(help = "sybil")
```

Get details of the usage of a particular function in *sybil* (e. g. `doubleGeneDel()`):

```
> help(doubleGeneDel)
```

Search through help files for a specific topic (e. g. "flux variability analysis"):

```
> help.search("flux variability analysis")
```

Open this vignette:

```
> vignette("sybil")
```

[3] http://CRAN.R-project.org/package=sybilSBML

4.2 Reading a model in tabular form

The package *sybil* can read metabolic network models written in tabular form as described in section 3.1. A reconstruction of the central metabolism of *E. coli* [Orth et al., 2010a, Palsson, 2006] is included as an example dataset. The example dataset consists of three files:

1. `Ec_core_desc.tsv` containing the model description,
2. `Ec_core_met.tsv` containing the metabolite list and
3. `Ec_core_react.tsv` containing the reaction list.

These files are located in the directory `extdata/` in the package *sybil*. The exact location of the files can be retrieved with the `system.file()` command:

```
> mp  <- system.file(package = "sybil", "extdata")
```

Now the model files can be read in by using the command `readTSVmod()`:

```
> mod <- readTSVmod(prefix = "Ec_core", fpath = mp, quoteChar = "\"")
```

```
model name:             Ecoli_core_model
number of compartments  2
                        C_c
                        C_e
number of reactions:    95
number of metabolites:  72
number of unique genes: 137
objective function:     +1 Biomass_Ecoli_core_w_GAM
```

If the fields in the input files for function `readTSVmod()` are quoted, argument `quoteChar` must be used. The value of `quoteChar` is passed to the argument `quote` of the R function `read.table()`. Argument `fpath` gets the path to the directory containing the model files. Argument `prefix` can be used, if the file names of the model files end like the file names used in the above example. All have the same base name `"Ec_core"` which is used for argument `prefix`. Function `readTSVmod()` now assumes, that the model files are named as follows:

- the model description file (if exists): `<prefix>_desc`,
- the list of metabolites (if exists): `<prefix>_met` and
- the list of reactions (must be there): `<prefix>_react`.

The file name suffix depends on the field delimiter. If the fields are tab-delimited, the default is `.tsv`. Function `readTSVmod()` returns an object of class `modelorg`. Models (instances of class `modelorg`, see section 5.1) can be converted to files in tabular form with the command `modelorg2tsv`:

```
> modelorg2tsv(mod, prefix = "Ec_core")
```

This will generate the three files shown in the list above (see also section 3.1). Load the example dataset included in *sybil*.

```
> data(Ec_core)
```

The example model is a 'ready to use' model, it contains a biomass objective function and an uptake of glucose [Orth et al., 2010a, Palsson, 2006]. It is the same model as used in the text files before.

4.3 Media conditions

The current set of exchange reactions in the model can be accessed with the function `findExchReact()`.

```
> ex <- findExchReact(Ec_core)
```

```
  #   pos.  reaction id          metabolite id         uptake     lb     ub
 [1]  20    EX_ac(e)             ac[e]                 FALSE       0    Inf
 [2]  21    EX_acald(e)          acald[e]              FALSE       0    Inf
 [3]  22    EX_akg(e)            akg[e]                FALSE       0    Inf
 [4]  23    EX_co2(e)            co2[e]                TRUE     -Inf    Inf
 [5]  24    EX_etoh(e)           etoh[e]               FALSE       0    Inf
 [6]  25    EX_for(e)            for[e]                FALSE       0    Inf
 [7]  26    EX_fru(e)            fru[e]                FALSE       0    Inf
 [8]  27    EX_fum(e)            fum[e]                FALSE       0    Inf
 [9]  28    EX_glc(e)            glc_D[e]              TRUE      -10    Inf
[10]  29    EX_gln_L(e)          gln_L[e]              FALSE       0    Inf
[11]  30    EX_glu_L(e)          glu_L[e]              FALSE       0    Inf
[12]  31    EX_h(e)              h[e]                  TRUE     -Inf    Inf
[13]  32    EX_h2o(e)            h2o[e]                TRUE     -Inf    Inf
[14]  33    EX_lac_D(e)          lac_D[e]              FALSE       0    Inf
[15]  34    EX_mal_L(e)          mal_L[e]              FALSE       0    Inf
[16]  35    EX_nh4(e)            nh4[e]                TRUE     -Inf    Inf
[17]  36    EX_o2(e)             o2[e]                 TRUE     -Inf    Inf
[18]  37    EX_pi(e)             pi[e]                 TRUE     -Inf    Inf
[19]  38    EX_pyr(e)            pyr[e]                FALSE       0    Inf
[20]  39    EX_succ(e)           succ[e]               FALSE       0    Inf

number of exchange reactions: 20
```

The subset of uptake reactions can be retrieved by method `uptReact`.

```
> upt <- uptReact(ex)
```

```
[1] "EX_co2(e)" "EX_glc(e)" "EX_h(e)"   "EX_h2o(e)" "EX_nh4(e)" "EX_o2(e)"
[7] "EX_pi(e)"
```

```
> ex[upt]
```

```
 #   pos.  reaction id           metabolite id          uptake    lb    ub
[1]  23    EX_co2(e)             co2[e]                 TRUE     -Inf   Inf
[2]  28    EX_glc(e)             glc_D[e]               TRUE      -10   Inf
[3]  31    EX_h(e)               h[e]                   TRUE     -Inf   Inf
[4]  32    EX_h2o(e)             h2o[e]                 TRUE     -Inf   Inf
[5]  35    EX_nh4(e)             nh4[e]                 TRUE     -Inf   Inf
[6]  36    EX_o2(e)              o2[e]                  TRUE     -Inf   Inf
[7]  37    EX_pi(e)              pi[e]                  TRUE     -Inf   Inf

number of exchange reactions: 7
```

Function `findExchReact()` returns an object of class `reactId_Exch` (extending class `reactId`) which can be used to alter reaction bounds with function `changeBounds()`. Make lactate the main carbon source instead of glucose:

```
> mod <- changeBounds(Ec_core, ex[c("EX_glc(e)", "EX_lac_D(e)")], lb = c(0, -10))
> findExchReact(mod)
```

```
  #   pos.  reaction id           metabolite id          uptake    lb    ub
 [1]  20    EX_ac(e)              ac[e]                  FALSE      0   Inf
 [2]  21    EX_acald(e)           acald[e]               FALSE      0   Inf
 [3]  22    EX_akg(e)             akg[e]                 FALSE      0   Inf
 [4]  23    EX_co2(e)             co2[e]                 TRUE    -Inf   Inf
 [5]  24    EX_etoh(e)            etoh[e]                FALSE      0   Inf
 [6]  25    EX_for(e)             for[e]                 FALSE      0   Inf
 [7]  26    EX_fru(e)             fru[e]                 FALSE      0   Inf
 [8]  27    EX_fum(e)             fum[e]                 FALSE      0   Inf
 [9]  28    EX_glc(e)             glc_D[e]               FALSE      0   Inf
[10]  29    EX_gln_L(e)           gln_L[e]               FALSE      0   Inf
[11]  30    EX_glu_L(e)           glu_L[e]               FALSE      0   Inf
[12]  31    EX_h(e)               h[e]                   TRUE    -Inf   Inf
[13]  32    EX_h2o(e)             h2o[e]                 TRUE    -Inf   Inf
[14]  33    EX_lac_D(e)           lac_D[e]               TRUE     -10   Inf
[15]  34    EX_mal_L(e)           mal_L[e]               FALSE      0   Inf
[16]  35    EX_nh4(e)             nh4[e]                 TRUE    -Inf   Inf
[17]  36    EX_o2(e)              o2[e]                  TRUE    -Inf   Inf
[18]  37    EX_pi(e)              pi[e]                  TRUE    -Inf   Inf
[19]  38    EX_pyr(e)             pyr[e]                 FALSE      0   Inf
[20]  39    EX_succ(e)            succ[e]                FALSE      0   Inf

number of exchange reactions: 20
```

4.4 Flux-balance analysis

Perform flux-balance analysis (FBA) by using method `optimizeProb` of class `modelorg`. Method `optimizeProb` performs flux-balance analysis [Edwards et al., 2002a, Orth et al., 2010b]. It returns a list containing the return value of the optimization process (`"ok"`), the solution status (`"stat"`), the value of the objective function after optimization (`"obj"`), the resulting flux distribution—the phenotype of the metabolic network—(`"fluxes"`) and results of pre- and post processing commands, if given (`"preP"` and `"postP"`). Also, a vector of integers will be returned (`"fldind"`). The flux value `fluxes[fldind[i]]` is the flux value of reaction `i` in the model (see section 4.5).

```
> optL <- optimizeProb(Ec_core, algorithm = "fba", retOptSol = FALSE)

$ok
[1] 0

$obj
[1] 0.8739215

$stat
[1] 5

$fluxes
 [1]  9.466331e-29  9.466331e-29 -4.985601e-28  6.007250e+00  6.007250e+00
 [6] -4.985601e-28  0.000000e+00  5.064376e+00  0.000000e+00  0.000000e+00
[11]  8.390000e+00  4.551401e+01  8.739215e-01 -2.280983e+01  6.007250e+00
[16]  4.359899e+01 -9.781875e-29  1.471614e+01  0.000000e+00  0.000000e+00
[21]  0.000000e+00  0.000000e+00  2.280983e+01  0.000000e+00  0.000000e+00
[26]  0.000000e+00  0.000000e+00 -1.000000e+01  0.000000e+00  0.000000e+00
[31]  1.753087e+01  2.917583e+01  0.000000e+00  0.000000e+00 -4.765319e+00
[36] -2.179949e+01 -3.214895e+00  0.000000e+00  0.000000e+00  7.477382e+00
[41]  0.000000e+00  0.000000e+00  0.000000e+00  0.000000e+00  0.000000e+00
[46]  5.064376e+00  0.000000e+00  4.959985e+00  1.602353e+01  1.000000e+01
[51]  2.234617e-01  0.000000e+00 -4.541857e+00  0.000000e+00  0.000000e+00
[56] -8.283040e-30  4.959985e+00 -2.917583e+01  6.007250e+00  0.000000e+00
[61] -9.781875e-29  0.000000e+00  0.000000e+00  5.064376e+00  0.000000e+00
[66]  0.000000e+00  3.853461e+01  0.000000e+00  4.765319e+00  2.179949e+01
[71]  9.282533e+00  7.477382e+00  0.000000e+00  4.860861e+00 -1.602353e+01
[76]  4.959985e+00 -1.471614e+01  3.214895e+00  2.504309e+00  0.000000e+00
[81]  0.000000e+00  8.835242e-29  1.758177e+00  1.577722e-29  2.678482e+00
[86] -2.281503e+00  0.000000e+00  2.505422e-27  5.064376e+00 -5.064376e+00
[91]  1.496984e+00  0.000000e+00  1.496984e+00  1.181498e+00  7.477382e+00

$preP
[1] NA
```

```
$postP
[1] NA

$fldind
 [1]  1  2  3  4  5  6  7  8  9 10 11 12 13 14 15 16 17 18 19 20 21 22 23 24 25
[26] 26 27 28 29 30 31 32 33 34 35 36 37 38 39 40 41 42 43 44 45 46 47 48 49 50
[51] 51 52 53 54 55 56 57 58 59 60 61 62 63 64 65 66 67 68 69 70 71 72 73 74 75
[76] 76 77 78 79 80 81 82 83 84 85 86 87 88 89 90 91 92 93 94 95
```

Perform FBA, return an object of class `optsol_optimizeProb` (extends class `optsol`, see section 5.2, this is the default behavior).

```
> opt <- optimizeProb(Ec_core, algorithm = "fba", retOptSol = TRUE)
```

```
solver:                                   glpkAPI
method:                                   simplex
algorithm:                                fba
number of variables:                      95
number of constraints:                    72
return value of solver:                   solution process was successful
solution status:                          solution is optimal
value of objective function (fba):        0.873922
value of objective function (model):      0.873922
```

The variable `opt` contains an object of class `optsol_optimizeProb`, a data structure storing all results of the optimization and providing methods to access the data (see section 5.2). Retrieve the value of the objective function after optimization.

```
> lp_obj(opt)
```

```
[1] 0.8739215
```

Translate the return and status codes of the optimization software into human readable strings.

```
> checkOptSol(opt)
```

```
Return code:
 Code    #       meaning
 0       1       solution process was successful

Solution status:
 Code    #       meaning
 5       1       solution is optimal
```

4.5 Minimize total flux

Usually, an FBA solution is not unique. There can be many equivalent flux distributions supporting the same objective value. A method to decide for one out of these solutions is to compute the flux distribution minimizing the total absolute flux (MTF) but still supporting the objective value of the FBA solution. At first, an objective value, for example calculated via FBA, is required:

```
> fba <- optimizeProb(Ec_core, algorithm = "fba")
```

Get the optimized value of the objective function:

```
> mod_obj(fba)
```

```
[1] 0.8739215
```

Now, the objective value of the solution in `fba` is used to compute a flux distribution with a minimized total absolute flux:

```
> mtf <- optimizeProb(Ec_core, algorithm = "mtf", wtobj = mod_obj(fba))
```

The value of the objective function for the MTF algorithm now is

```
> lp_obj(mtf)
```

```
[1] 518.4221
```

which is the minimized sum of all absolute flux values. The number of variables of the MTF problem is three times the number of the variables of the FBA solution

```
> nvar(fluxdist(fba))
```

```
[1] 95
```

```
> nvar(fluxdist(mtf))
```

```
[1] 285
```

which is due to the different formulations of the two optimization problems. Consult the documentation of class `sysBiolAlg` (section 5.4) for more detailed information on the algorithms.

```
> help("sysBiolAlg_fba-class")
> help("sysBiolAlg_mtf-class")
```

There are also shortcuts available:

```
> ?fba
> ?mtf
```

Method getFluxDist can be used to get the flux distribution of the MTF solution; the values of the variables corresponding to the reactions in the metabolic network.

```
> fl <- getFluxDist(mtf)
> length(fl)
```

```
[1] 95
```

The flux distribution of the exchange reactions can be retrieved in a similar way (variable ex contains the exchange reactions of the *E. coli* model, see section 4.3).

```
> fd <- getFluxDist(mtf, ex)
```

```
     EX_ac(e)   EX_acald(e)     EX_akg(e)     EX_co2(e)    EX_etoh(e)
-5.730253e-15  0.000000e+00  0.000000e+00  2.280983e+01  0.000000e+00
    EX_for(e)     EX_fru(e)     EX_fum(e)     EX_glc(e)   EX_gln_L(e)
 0.000000e+00  0.000000e+00  0.000000e+00 -1.000000e+01  0.000000e+00
  EX_glu_L(e)       EX_h(e)     EX_h2o(e)   EX_lac_D(e)   EX_mal_L(e)
 0.000000e+00  1.753087e+01  2.917583e+01  0.000000e+00  0.000000e+00
    EX_nh4(e)      EX_o2(e)      EX_pi(e)     EX_pyr(e)    EX_succ(e)
-4.765319e+00 -2.179949e+01 -3.214895e+00  0.000000e+00  0.000000e+00
```

```
> getNetFlux(fd)
```

```
uptake reaction rates (absolute values):
 EX_glc(e)          10.000000
 EX_nh4(e)           4.765319
 EX_o2(e)           21.799493
 EX_pi(e)            3.214895

excretion reaction rates (absolute values):
 EX_co2(e)          22.809833
 EX_h(e)            17.530865
 EX_h2o(e)          29.175827

unused exchange reactions [abs(rate) < 1e-06]:
 [1] "EX_ac(e)"    "EX_acald(e)" "EX_akg(e)"   "EX_co2(e)"   "EX_etoh(e)"
 [6] "EX_for(e)"   "EX_fru(e)"   "EX_fum(e)"   "EX_glc(e)"   "EX_gln_L(e)"
[11] "EX_glu_L(e)" "EX_h(e)"     "EX_h2o(e)"   "EX_lac_D(e)" "EX_mal_L(e)"
[16] "EX_nh4(e)"   "EX_o2(e)"    "EX_pi(e)"    "EX_pyr(e)"   "EX_succ(e)"
```

Function getNetFlux() the absolute flux values of the exchange reactions grouped by flux direction. The value of the objective function given in the model (here: biomass production) can be accessed by method mod_obj:

```
> mod_obj(mtf)
```

```
[1] 0.8739215
```

which is of course the same value as for the FBA algorithm.

4.6 Genetic perturbations

To compute the metabolic phenotypes of *in silico* knock-out mutants, argument `gene` of method `optimizeProb` can be used.

```
> ko <- optimizeProb(Ec_core, gene = "b2276", lb = 0, ub = 0)

solver:                                   glpkAPI
method:                                   simplex
algorithm:                                fba
number of variables:                      95
number of constraints:                    72
return value of solver:                   solution process was successful
solution status:                          solution is optimal
value of objective function (fba):        0.211663
value of objective function (model):      0.211663
```

Argument `gene` gets a character vector of gene locus tags present in the used model. The flux boundaries of reactions affected by the genes given in argument `gene` will be set to the values of arguments `lb` and `ub`. If both arguments are set to zero, no flux through the affected reactions is allowed.

4.6.1 Minimization of metabolic adjustment (MOMA)

The default algorithm used by method `optimizeProb` is FBA [Edwards et al., 2002a, Orth et al., 2010b], implying the assumption, that the phenotype of the mutant metabolic network is independent of the wild-type phenotype. An alternative is the MOMA algorithm described in Segrè et al. [2002] minimizing the hamiltonian distance of the wild-type phenotype and the mutant phenotype (argument `algorithm = "lmoma"` computes a linearized version of the MOMA algorithm; `algorithm = "moma"` runs the quadratic formulation).

```
> ko <- optimizeProb(Ec_core, gene = "b2276", lb = 0, ub = 0,
+                    algorithm = "lmoma", wtflux = getFluxDist(mtf))

solver:                                   glpkAPI
method:                                   simplex
algorithm:                                lmoma
number of variables:                      380
number of constraints:                    262
return value of solver:                   solution process was successful
solution status:                          solution is optimal
value of objective function (lmoma):      424.324062
value of objective function (model):      0.081964
```

Table 2: Functions used to simulate multiple *in silico* knock-out mutants.

function	purpose
`oneGeneDel()`	single gene knock-outs
`doubleGeneDel()`	pairwise gene knock-outs
`geneDeletion()`	simultaneous deletion of n genes

The variable `ko` contains the solution of the linearized version of the MOMA algorithm. A wild-type flux distribution can be set via argument `wtflux`, here, the flux distribution computed through the MTF algorithm was used. If argument `wtflux` is not set, a flux distribution based on FBA will be used.

4.6.2 Regulatory on/off minimization (ROOM)

Another alternative is the ROOM algorithm (regulatory on/off minimization) described in Shlomi et al. [2005]. Set argument `algorithm` to `room` in order to run ROOM.

```
> ko <- optimizeProb(Ec_core, gene = "b2276", lb = 0, ub = 0,
+                    algorithm = "room", wtflux = getFluxDist(mtf),
+                    solverParm = list(PRESOLVE = GLP_ON))
```

ROOM is a mixed integer programming problem which requires for GLPK to switch on the pre-solver. See section 4.14 for more information on setting parameters to the mathematical programming software.

4.6.3 Multiple knock-outs

Method `optimizeProb` can be used to study the metabolic phenotype of one knock-out mutant. The purpose of functions `oneGeneDel()`, `doubleGeneDel()` and `geneDeletion()` is the simulation of multiple *in silico* knock-outs (see table 2). Function `oneGeneDel()` simulates all possible single gene knock-out mutants in a metabolic model (default algorithm is FBA).

```
> opt <- oneGeneDel(Ec_core)

|            :            |            :           | 100 %
|==================================================| :-)
solver:                                   glpkAPI
method:                                   simplex
algorithm:                                fba
number of variables:                      95
number of constraints:                    72
number of problems to solve:              137
number of successful solution processes:  137
```

The function `oneGeneDel` gets an argument `geneList`, a character vector containing the gene id's to knock out. If `geneList` is missing, all genes are taken into account. The example model contains 137 independent genes, so 137 optimizations will be performed.

The result stored in the variable `opt` is an object of class `optsol_geneDel`, extending class `optsol_optimizeProb` (see section 4.4). Method `checkOptSol` gives an overview about the results and status of the optimizations. Additionally, class `optsol` contains a method `plot`, plotting a histogram of the values of the objective function given in the model after optimization (section 5.2).

```
> checkOptSol(opt)

Return code:
 Code    #       meaning
 0       137     solution process was successful

Solution status:
 Code    #       meaning
 4       2       no feasible solution exists
 5       135     solution is optimal

137 optimizations were performed.
```

Plot the histogram:

```
> plot(opt, nint = 20)
```

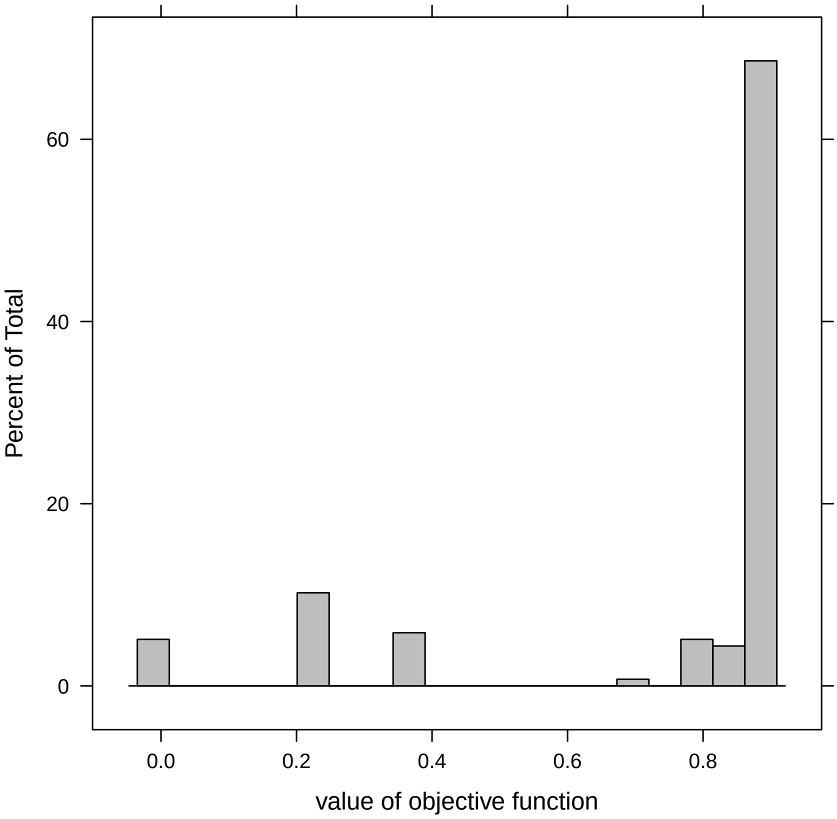

Argument `algorithm` can be used here to use MOMA to compute the mutant flux distributions. Additionally, a wild-type solution can be provided.

```
> opt <- oneGeneDel(Ec_core, algorithm = "lmoma", wtflux = getFluxDist(mtf))

|            :            |            :            | 100 %
|==================================================| :-)

> checkOptSol(opt)

Return code:
 Code    #       meaning
 0       137     solution process was successful

Solution status:
 Code    #       meaning
 4       2       no feasible solution exists
 5       135     solution is optimal

137 optimizations were performed.
```

```
> plot(opt, nint = 20)
```

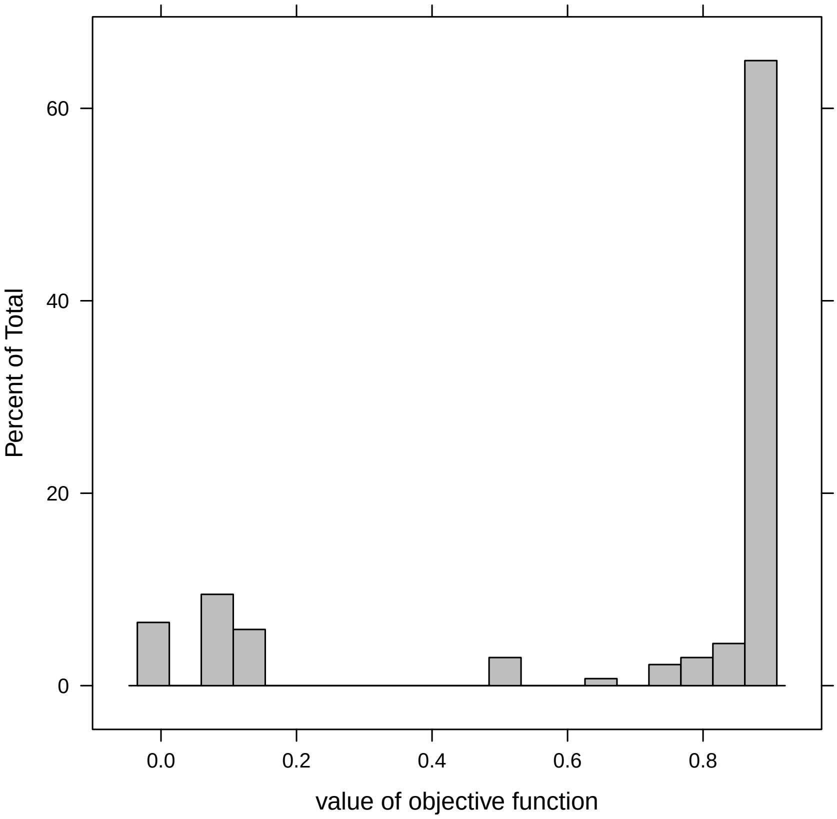

In order to perform all possible double-knock-out mutants, or n-knock-out mutants, the function `geneDeletion` can be used. Perform single gene deletions (in principle the same as before with `oneGeneDel`).

```
> opt <- geneDeletion(Ec_core)

|               :               |               :               | 100 %
|===================================================| :-)
```

Compute all double-knock-out mutants and all triple-knock-out mutants

```
> opt2 <- geneDeletion(Ec_core, combinations = 2)
> opt3 <- geneDeletion(Ec_core, combinations = 3)
```

which will result in 9317 optimizations for double-knock-outs and 419 221 Optimizations for triple-knock-outs using the metabolic model of the core energy metabolism of *E. coli*. This model contains 137 genes.

4.7 Flux variability analysis

The function `fluxVar` performs a flux variability analysis with a given model [Mahadevan and Schilling, 2003]. The minimum and maximum flux values for each reaction in the model are calculated, which still support a certain percentage of a given optimal functional state Z_{opt}.

```
> opt <- fluxVar(Ec_core, percentage = 80, verboseMode = 0)
> plot(opt)
```

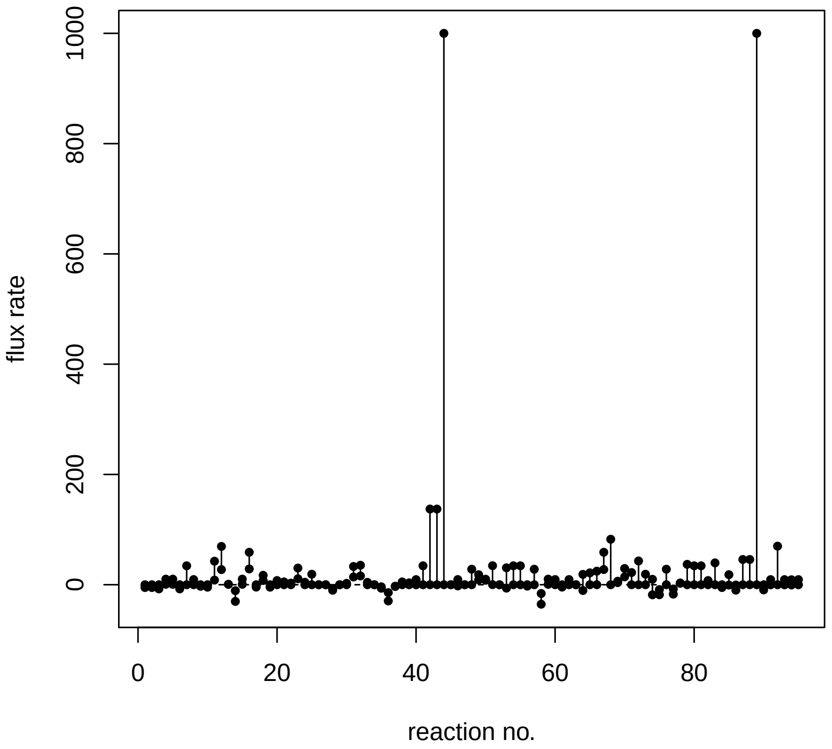

4.8 Robustness analysis

The function `robAna` performs a robustness analysis with a given model. The flux of a control reaction will be varied stepwise between the maximum and minimum value the flux of the control reaction can reach [Palsson, 2006]. The example below shows a flux variability analysis based upon the metabolic model of the core energy metabolism of *E. coli* using the exchange flux of oxygen as control reaction.

```
> opt <- robAna(Ec_core, ctrlreact = "EX_o2(e)", verboseMode = 0)
> plot(opt)
```

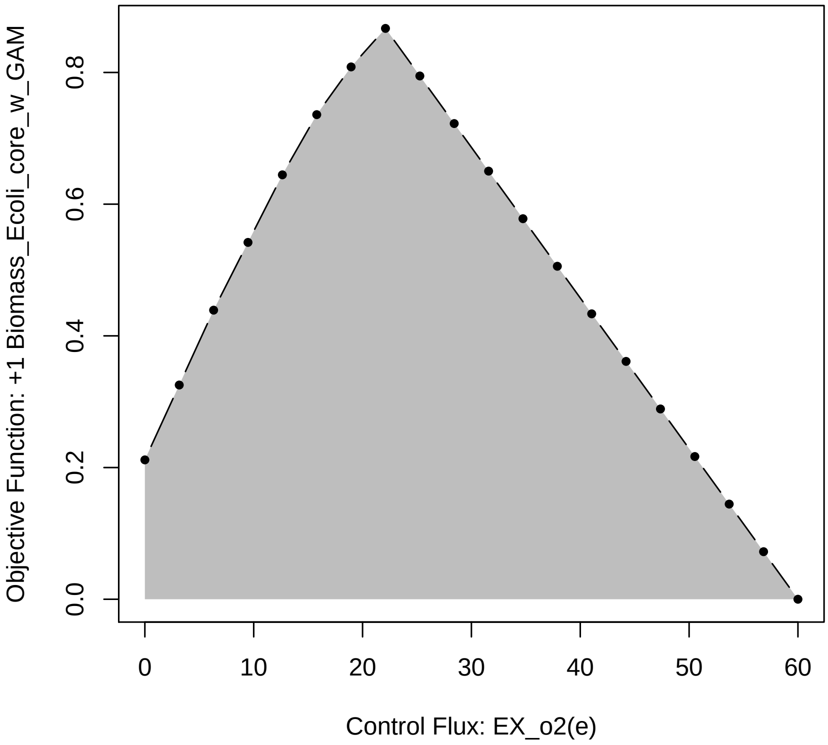

4.9 Phenotypic phase plane analysis

The function phpp performs a phenotypic phase plane analysis [Edwards et al., 2001, 2002b] with a given model. The flux of two control reactions will be varied stepwise between a given maximum and minimum value. The example below shows a phenotypic phase plane analysis based upon the metabolic model of the core energy metabolism of *E. coli* using the exchange fluxes of oxygen and succinate as control reactions. First, the uptake of glucose is switched off.

```
> Ec_core_wo_glc <- changeUptake(Ec_core, off = "glc_D[e]")
> opt <- phpp(Ec_core_wo_glc,
+             ctrlreact = c("EX_succ(e)", "EX_o2(e)"),
+             redCosts = TRUE,
+             numP = 25,
+             verboseMode = 0)
> plot(opt)
```

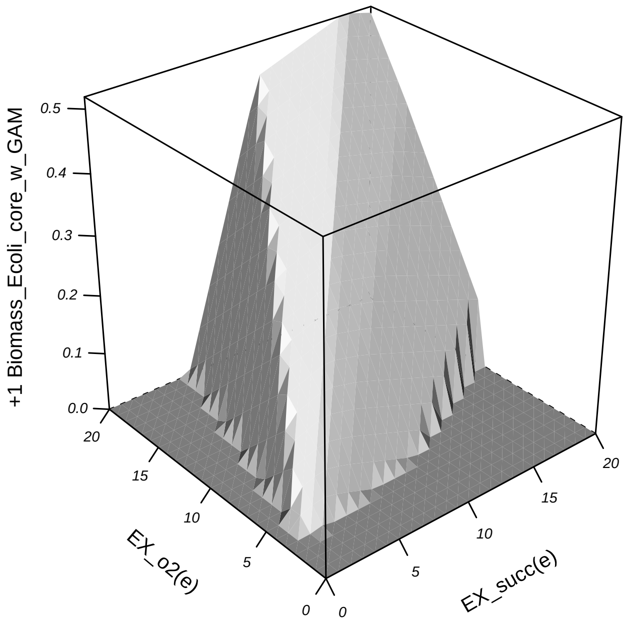

Plot reduced costs of succinate import flux and oxygen import flux.

```
> plot(opt, "EX_succ(e)")
> plot(opt, "EX_o2(e)")
```

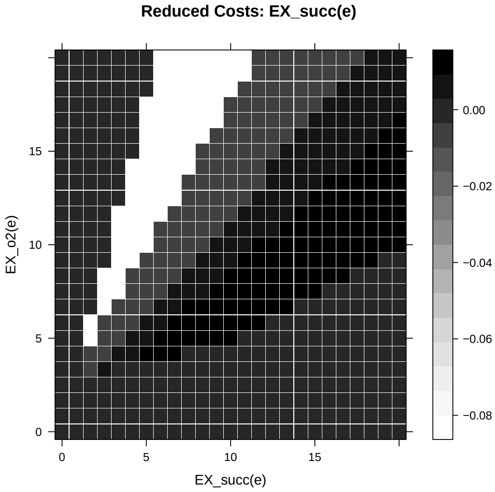

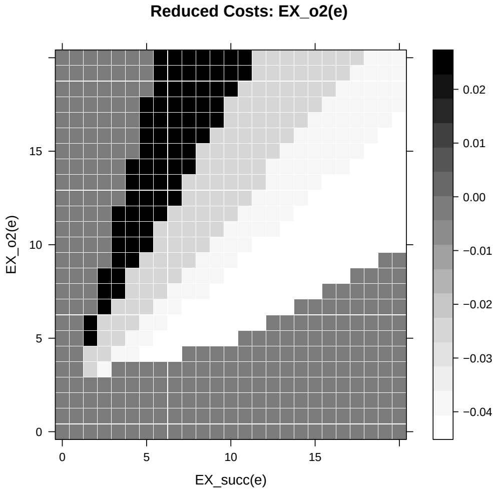

4.10 Summarizing simulation results

Each simulation generates an object of class `optsol` containing all the results of the optimizations. In order to get a quick overview of the results, the function `summaryOptsol()` can be used. At first, let's compute all single gene knock-outs of the metabolic model of the core energy metabolism of *E. coli*:

```
> opt <- oneGeneDel(Ec_core, algorithm = "fba", fld = "all")

|            :            |            :            | 100 %
|=====================================================| :-)
```

Generate a summary:

```
> sum <- summaryOptsol(opt, Ec_core)

flux distribution:
 number of elements:          13015
 number of zeros:             5986
 number of non zero elements: 7029

exchange metabolites:
 [1] ac[e]    acald[e] akg[e]   co2[e]   etoh[e]  for[e]   fru[e]   fum[e]
 [9] glc_D[e] gln_L[e] glu_L[e] h[e]     h2o[e]   lac_D[e] mal_L[e] nh4[e]
[17] o2[e]    pi[e]    pyr[e]   succ[e]

substrates (-) and products (+):
 use method 'printExchange()'

limiting reactions:
 there is about one limiting reaction per optimization;
 use method 'printReaction()' to see more details

optimal values of model objective function:
   Min. 1st Qu.  Median    Mean 3rd Qu.    Max.
 0.0000  0.8143  0.8739  0.7263  0.8739  0.8739

summary of optimization process:
Return code:
 Code    #       meaning
 0       137     solution process was successful

Solution status:
 Code    #       meaning
 4       2       no feasible solution exists
 5       135     solution is optimal
```

```
137 optimizations were performed.
```

The function `summaryOptsol()` returns an object of class `optsolSummary` and needs the object of class `optsol` (results of simulations) and the corresponding object of class `modelorg` (the entire metabolic network). The generated object of class `summaryOptsol` contains some information about the flux distribution, substrates, products and limiting reactions.

The method `printExchange()` prints a subset of the flux distribution for the exchange reactions in the model. Each column represents the environment of one optimization. The symbol `"-"` indicates that the corresponding metabolite is imported (is a substrate); the symbol `"+"` indicates, that the corresponding metabolite excreted (is a product).

```
> printExchange(sum, j = c(1:50), dense = TRUE)
```

```
     ac[e]   +            ++++++++ +
  acald[e]
    akg[e]
    co2[e] ++ ++++++++++++--------+++++++++++++++++++++++++++
   etoh[e]   +
    for[e]   +      +     ++++++++
    fru[e]
    fum[e]
  glc_D[e] --------------------------------------------------
  gln_L[e]
  glu_L[e]
      h[e] +++++++++++++++++++++++++++++++ ++++++++++++++  ++
    h2o[e] ++-+++++++++++++++++++++++++++++++++++++++++++++++
  lac_D[e]
  mal_L[e]
    nh4[e] ------------------------ ------ --------------  --
     o2[e] -- -----------------------------------------------
     pi[e] ------------------------ ------ --------------  --
    pyr[e]
   succ[e]
```

4.11 Parallel computing

The package *sybil* provides basic support for the R-package *parallel* in function `multidel()`. The following example shows the computation of all possible triple-knock-out mutants using the model of the core energy metabolism of *E. coli.* The set of genes included in the analysis will be reduced to genes, which are not lethal. A gene i is considered as "lethal", if in a single-gene-knockout the deletion of gene i results in a maximum growth ratio of zero.

```
> ref    <- optimizeProb(Ec_core)
> opt    <- oneGeneDel(Ec_core)

|            :            |            :            | 100 %
|===================================================| :-)

> let    <- lethal(opt, wt = mod_obj(ref))
> nletid <- c(1:length(allGenes(Ec_core)))[! let]
```

At first, a wild-type maximum growth rate is computed and stored in the variable `ref`. Then, all single-gene knock-outs are computed. The variable `let` contains pointers to the gene id's of genes, who's deletion is lethal. The variable `nletid` contains pointers to the gene id's of all genes, except for the lethal ones.

```
> gmat <- combn(nletid, 3)
```

The variable `gmat` now contains a matrix with three rows, each column is one combination of three values in `nletid`; one set of genes to knock-out in one step.

```
> opt <- multiDel(Ec_core, nProc = 4, todo = "geneDeletion", del1 = gmat)
```

The function `multiDel` performs a `geneDeletion` with the model `Ec_core` on four CPU's (argument `nProc`) on a shared memory machine. Argument `del1` is the matrix containing the sets of genes to delete. This matrix will be split up in smaller sub-matrices all having about the same number of columns and three rows. The sub-matrices are passed to `geneDeletion` and are processed on separate cores in parallel. The resulting variable `opt` now contains a list of four objects of class `optsol_genedel`. Use `mapply` to access the values stored in the `optsol` objects.

```
> mapply(checkOptSol, opt)
```

4.12 Interacting with the optimization process

Method `optimizeProb` provides a basic mechanism to run commands before and or after solving the optimization problem. In order to retrieve the reduced costs after the optimization, use argument `poCmd`.

```
> opt <- optimizeProb(Ec_core, poCmd = list("getRedCosts"))
> postProc(opt)

An object of class "ppProc"
Slot "cmd":
[[1]]
[1] "getRedCosts(LP_PROB)"


Slot "pa":
```

```
[[1]]
 [1]  0.000000000  0.000000000  0.000000000  0.000000000  0.000000000
 [6]  0.000000000  0.000000000  0.000000000  0.000000000  0.000000000
[11] -0.005092486  0.000000000  0.000000000  0.000000000  0.000000000
[16]  0.000000000  0.000000000  0.000000000  0.000000000 -0.022916187
[21] -0.034374280 -0.061109831  0.000000000 -0.039466766 -0.007638729
[26] -0.091664746 -0.045832373 -0.091664746 -0.070021681 -0.068748560
[31]  0.000000000  0.000000000 -0.040739887 -0.045832373  0.000000000
[36]  0.000000000  0.000000000 -0.034374280 -0.052197981  0.000000000
[41] -0.005092486 -0.001273121  0.000000000  0.000000000  0.000000000
[46]  0.000000000  0.000000000  0.000000000  0.000000000  0.000000000
[51]  0.000000000  0.000000000  0.000000000 -0.005092486 -0.005092486
[56]  0.000000000  0.000000000  0.000000000  0.000000000  0.000000000
[61]  0.000000000 -0.001273121  0.000000000  0.000000000 -0.005092486
[66] -0.003819364  0.000000000 -0.001273121  0.000000000  0.000000000
[71]  0.000000000  0.000000000  0.000000000  0.000000000  0.000000000
[76]  0.000000000  0.000000000  0.000000000  0.000000000 -0.005092486
[81] -0.005092486  0.000000000  0.000000000  0.000000000  0.000000000
[86]  0.000000000 -0.003819364  0.000000000  0.000000000  0.000000000
[91]  0.000000000 -0.001273121  0.000000000  0.000000000  0.000000000


Slot "ind":
integer(0)
```

4.13 Optimization software

To solve optimization problems, GLPK[4], IBM ILOG CPLEX[5], COIN-OR Clp[6] or lp_solve[7] can be used (package *sybilGUROBI* providing support for Gurobi optimization[8] is available on request). All functions performing optimizations, get the arguments `solver` and `method`. The first setting the desired solver and the latter setting the desired optimization algorithm. Possible values for the argument `solver` are:

- `"glpkAPI"`, which is the default,
- `"cplexAPI"`,
- `"clpAPI"` or

[4]Andrew Makhorin: GNU Linear Programming Kit, version 4.42 or higher
`http://www.gnu.org/software/glpk/glpk.html`

[5]IBM ILOG CPLEX version 12.2 (or higher) from the IBM Academic Initiative
`https://www.ibm.com/developerworks/university/academicinitiative/`

[6]COIN-OR linear programming version 1.12.0 or higher `https://projects.coin-or.org/Clp`

[7]lp_solve via R-package *lpSolveAPI* version 5.5.2.0-5 or higher
`http://lpsolve.sourceforge.net/5.5/index.htm`

[8]`http://www.gurobi.com`

- "lpSolveAPI".

Perform FBA, using GLPK as solver and "simplex exact" as algorithm.

```
> optimizeProb(Ec_core, method = "exact")
```

Perform FBA, using IBM ILOG CPLEX as solver and "dualopt" as algorithm.

```
> optimizeProb(Ec_core, solver = "cplexAPI", method = "dualopt")
```

The R-packages *glpkAPI*, *clpAPI* and *cplexAPI* provide access to the C-API of the corresponding optimization software. They are also available from CRAN[1].

4.14 Setting parameters to the optimization software

All functions performing optimizations can handle the argument `solverParm` getting a list or data frame containing parameters used by the optimization software.

4.14.1 GLPK

For available parameters used by GLPK, see the GLPK and the *glpkAPI* documentation.

```
> opt <- oneGeneDel(Ec_core,
+                   solverParm = list(TM_LIM = 1000,
+                                     PRESOLVE = GLP_ON))
```

The above command performs a single gene deletion experiment (see section 4.6.3), sets the time limit for each optimization to one second and does pre-solving in each optimization.

4.14.2 IBM ILOG CPLEX

For available parameters used by IBM ILOG CPLEX, see the IBM ILOG CPLEX and the *cplexAPI* documentation.

```
> opt <- optimizeProb(Ec_core,
+                     solverParm = list(CPX_PARAM_SCRIND = CPX_ON,
+                                       CPX_PARAM_EPRHS = 1E-09),
+                     solver = "cplexAPI")
```

The above command performs FBA, sets the messages to screen switch to "on" and sets the feasibility tolerance to 10^{-9}.

4.14.3 COIN-OR Clp

At the time of writing, it is not possible to set any parameters when using COIN-OR Clp.

Table 3: Available parameters in *sybil* and their default values.

parameter name	default value
SOLVER	glpkAPI
METHOD	simplex
SOLVER_CTRL_PARM	as.data.frame(NA)
ALGORITHM	fba
TOLERANCE	1E-6
MAXIMUM	1000
OPT_DIRECTION	max
PATH_TO_MODEL	.

4.14.4 lpSolveAPI

See the *lpSolveAPI* documentation for parameters for `lp_solve`.

```
> opt <- optimizeProb(Ec_core,
+                     solverParm = list(verbose = "full",
+                                       timeout = 10),
+                     solver = "lpSolveAPI")
```

The above command performs FBA, sets the verbose mode to "full" and sets the timeout to ten seconds.

4.15 Setting parameters in sybil

Parameters to *sybil* can be set using the function `SYBIL_SETTINGS`. Parameter names and their default values are shown in table 3, all possible values are described in the `SYBIL_SETTINGS` documentation.

```
> help(SYBIL_SETTINGS)
```

The function `SYBIL_SETTINGS` gets at most two arguments:

```
> SYBIL_SETTINGS("parameter name", value)
```

the first one giving the name of the parameter to set (as character string) and the second one giving the desired value. If `SYBIL_SETTINGS` is called with only one argument

```
> SYBIL_SETTINGS("parameter name")
```

the current setting of `"parameter name"` will be returned. All parameters and their values can be achieved by calling `SYBIL_SETTINGS` without any argument.

```
> SYBIL_SETTINGS()
```

4.15.1 Solver software specific

The two parameters `SOLVER` and `METHOD` depend on each other, e. g. the method called `simplex` is only available when `glpkAPI` is used as solver software. Each solver has its own specific set of methods available in order to solve optimization problems. If one changes the parameter `SOLVER` to, let's say `cplexAPI`, the parameter `METHOD` will automatically be adjusted to the default method used by `cplexAPI`. Set parameter solver to IBM ILOG CPLEX for every optimization:

```
> SYBIL_SETTINGS("SOLVER", "cplexAPI", loadPackage = FALSE)
```

Now, IBM ILOG CPLEX is used as default solver e. g. in `optimizeProb` or `oneGeneDel`, and parameter `METHOD` has changed to the default method in *cplexAPI*. Setting argument `loadPackage` to `FALSE` prevents loading the API package. Get the current setting for `Method`:

```
> SYBIL_SETTINGS("METHOD")
```

```
[1] "lpopt"
```

Reset the solver to `glpkAPI`:

```
> SYBIL_SETTINGS("SOLVER", "glpkAPI")
```

Now, the default method again is `simplex`

```
> SYBIL_SETTINGS("METHOD")
```

```
[1] "simplex"
```

It is not possible to set a wrong method for a given solver. If the desired method is not available, always the default method is used. Parameters to the solver software (parameter `SOLVER_CTRL_PARM`) must be set as `list` or `data.frame` as described in section 4.14.

4.15.2 Analysis specific

The parameter `ALGORITHM` controls the way gene deletion analysis will be performed. The default setting `"fba"` will use flux-balance analysis (FBA) as described in Edwards et al. [2002a] and Orth et al. [2010b]. Setting this parameter to `"lmoma"`, results in a linearized version of the MOMA algorithm described in Segrè et al. [2002] ("moma" will run the original version). The linearized version of MOMA, like it is implemented in the COBRA Toolbox [Becker et al., 2007, Schellenberger et al., 2011], can be used in functions like `oneGeneDel()` via the Boolean argument `COBRAflag`. Setting the parameter `"ALGORITHM"` to `"room"` will run a regulatory on/off minimization as described in Shlomi et al. [2005]. See also section 4.6 for details on gene deletion analysis.

5 Central data structures

5.1 Class modelorg

The class `modelorg` is the core datastructure to represent a metabolic network, in particular the stoichiometric matrix S. An example (E. coli core flux by [Palsson, 2006]) is shipped within *sybil* and can be loaded this way:

```
> data(Ec_core)
> Ec_core

model name:             Ecoli_core_model
number of compartments  2
                        C_c
                        C_e
number of reactions:    95
number of metabolites:  72
number of unique genes: 137
objective function:     +1 Biomass_Ecoli_core_w_GAM
```

The generic method `show` displays a short summary of the parameters of the metabolic network. See

```
> help("modelorg")
```

for the list of available methods. All slots of an object of class `modelorg` are accessible via setter and getter methods having the same name as the slot. For example, slot `react_num` contains the number of reactions in the model (equals the number of columns in S). Access the number of reactions in the *E. coli* model.

```
> react_num(Ec_core)

[1] 95
```

Get all reaction is's:

```
> id <- react_id(Ec_core)
```

Change a reaction id:

```
> react_id(Ec_core)[13] <- "biomass"
```

Plot an image of the stoichiometric matrix S:

```
> cg <- gray(0:8/8)
> image(S(Ec_core), col.regions = c(cg, rev(cg)))
```

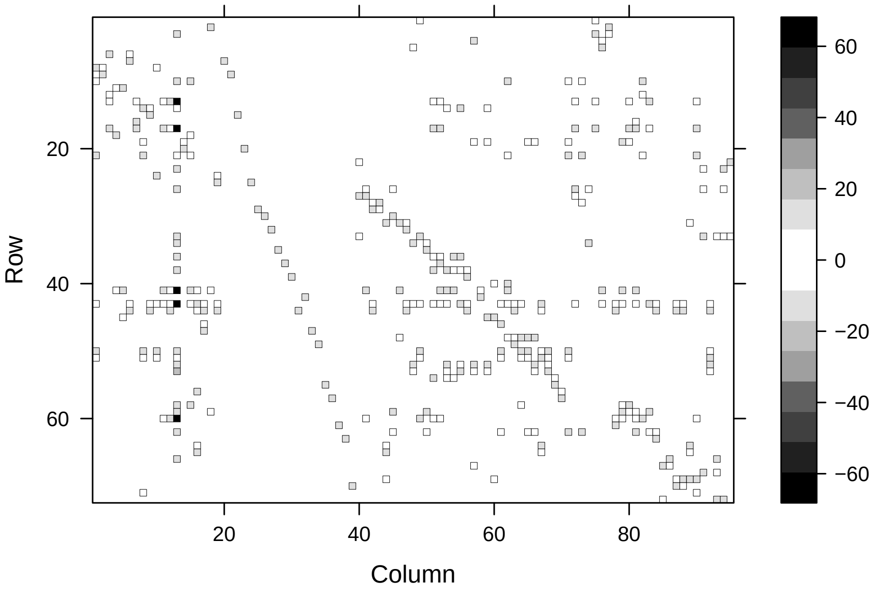

Dimensions: 72 x 95

Matrices in objects of class `modelorg` are stored in formats provided by the *Matrix*-package[9].

Objects of class `modelorg` can easily be created. Sources are common file formats like tab delimited files from the BiGG database [Schellenberger et al., 2010][10] or SBML files (with package *sybilSBML*[3]). See section 3 on page 2 about supported file formats and their description. Read a reaction list generated from the BiGG database:

```
> mod <- readTSVmod(reactList = "reactionList.txt")
```

Here, `"reactionList.txt"` is an from BiGG database exported reaction list. Usually, these files do neither contain an objective function, nor upper and lower bounds on the reaction rates. They need to be added to the returned object of class `modelorg` using the methods `obj_coef<-`, `lowbnd<-` and `uppbnd<-`, or by adding the columns `obj_coef`, `lowbnd` and `uppbnd` to the input file.

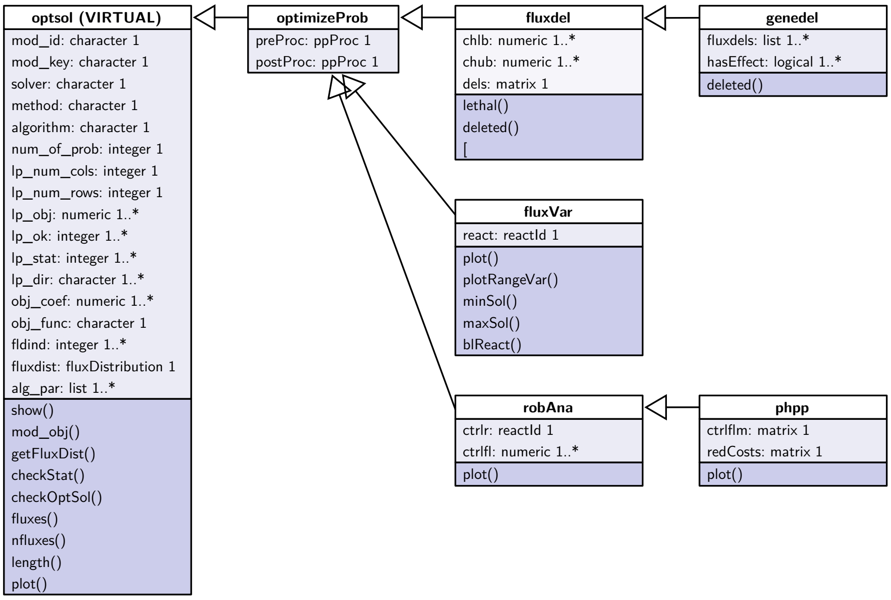

Figure 2: UML representation of class `optsol`.

5.2 Class optsol

The derived classes of class `optsol` (optimization solution) are used to store information and results from various optimization problems and their biological relation. See

```
> help("optsol")
```

for the list of available methods to access the data (figure 2). A simple demonstration would be:

```
> os <- optimizeProb(Ec_core)
```

```
solver:                                   glpkAPI
method:                                   simplex
algorithm:                                fba
number of variables:                      95
number of constraints:                    72
return value of solver:                   solution process was successful
solution status:                          solution is optimal
value of objective function (fba):        0.873922
value of objective function (model):      0.873922
```

```
> is(os)
```

[9]http://CRAN.R-project.org/package=Matrix
[10]`http://bigg.ucsd.edu`

```
[1] "optsol_optimizeProb" "optsol"
```

Retrieve objective value.

```
> lp_obj(os)
```

```
[1] 0.8739215
```

Retrieve flux distribution.

```
> getFluxDist(os)
```

```
            1             2             3             4             5
 9.466331e-29  9.466331e-29 -4.985601e-28  6.007250e+00  6.007250e+00
            6             7             8             9            10
-4.985601e-28  0.000000e+00  5.064376e+00  0.000000e+00  0.000000e+00
           11            12            13            14            15
 8.390000e+00  4.551401e+01  8.739215e-01 -2.280983e+01  6.007250e+00
           16            17            18            19            20
 4.359899e+01 -9.781875e-29  1.471614e+01  0.000000e+00  0.000000e+00
           21            22            23            24            25
 0.000000e+00  0.000000e+00  2.280983e+01  0.000000e+00  0.000000e+00
           26            27            28            29            30
 0.000000e+00  0.000000e+00 -1.000000e+01  0.000000e+00  0.000000e+00
           31            32            33            34            35
 1.753087e+01  2.917583e+01  0.000000e+00  0.000000e+00 -4.765319e+00
           36            37            38            39            40
-2.179949e+01 -3.214895e+00  0.000000e+00  0.000000e+00  7.477382e+00
           41            42            43            44            45
 0.000000e+00  0.000000e+00  0.000000e+00  0.000000e+00  0.000000e+00
           46            47            48            49            50
 5.064376e+00  0.000000e+00  4.959985e+00  1.602353e+01  1.000000e+01
           51            52            53            54            55
 2.234617e-01  0.000000e+00 -4.541857e+00  0.000000e+00  0.000000e+00
           56            57            58            59            60
-8.283040e-30  4.959985e+00 -2.917583e+01  6.007250e+00  0.000000e+00
           61            62            63            64            65
-9.781875e-29  0.000000e+00  0.000000e+00  5.064376e+00  0.000000e+00
           66            67            68            69            70
 0.000000e+00  3.853461e+01  0.000000e+00  4.765319e+00  2.179949e+01
           71            72            73            74            75
 9.282533e+00  7.477382e+00  0.000000e+00  4.860861e+00 -1.602353e+01
           76            77            78            79            80
 4.959985e+00 -1.471614e+01  3.214895e+00  2.504309e+00  0.000000e+00
           81            82            83            84            85
```

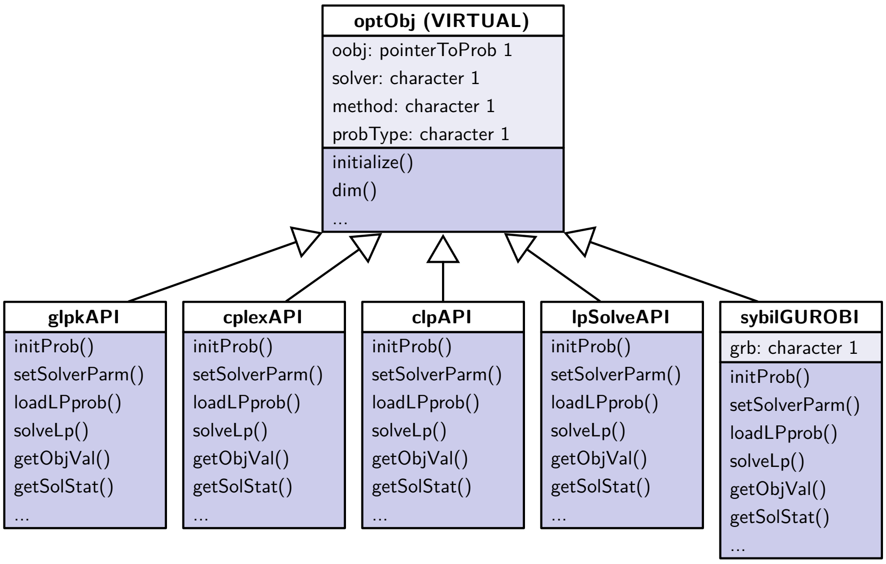

Figure 3: UML representation of class `optObj`.

```
 0.000000e+00  8.835242e-29  1.758177e+00  1.577722e-29  2.678482e+00
           86            87            88            89            90
-2.281503e+00  0.000000e+00  2.505422e-27  5.064376e+00 -5.064376e+00
           91            92            93            94            95
 1.496984e+00  0.000000e+00  1.496984e+00  1.181498e+00  7.477382e+00
```

5.3 Class optObj

The class `optObj` is *sybil*'s internal representation of an optimization problem (figure 3). Objects of this class harbor four slots: i) `oobj`: a pointer to the C-structure of the problem object (depending on the solver software), ii) `solver`: the name of the solver, iii) `method`: the name of the optimization method used by the solver and iv) `probType`: single character string, describing the problem type (e. g. `lp`: linear programming, or `mip`: mixed integer programming).

The package *sybil* provides several functions to alter the problem object. Each function takes care of the special needs of every supported solver. The following example should illustrate the purpose of class `optObj`. Consider a linear programming problem, here written in lp formatted file format:

```
Maximize
  obj: + x_1 + x_2
Subject To
  r_1: + 0.5 x_1 + x_2 <= 4.5
  r_2: + 2   x_1 + x_2 <= 9
Bounds
```

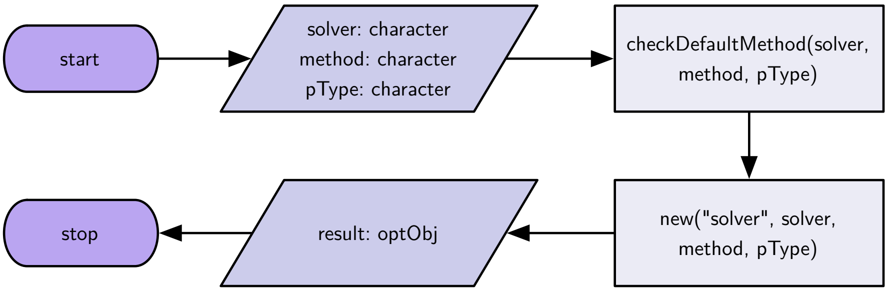

Figure 4: Work flow of the constructor function `optObj()`.

```
0 <= x_1 <= 1000
0 <= x_2 <= 1000
```

In order to solve this lp problem with *sybil*, an object of class `optObj` has to be created. The constructor function has the same name as the class it builds.

```
> lp <- optObj(solver = "glpkAPI", method = "exact")
```

```
linear programming problem object
solver: glpkAPI
method: exact
problem is not initialized
```

The first argument is the used solver software, in this case it is GLPK. The second optional argument gives the method, how the solver software has to solve the problem. Here, it is the simplex exact algorithm of GLPK. The constructor function `optObj()` (figure 4) returns an object of class `optObj_glpkAPI` in this case. This class enables *sybil* to communicate with the GLPK software. The constructor function `optObj()` calls the function `checkDefaultMethod()` which tries to load the specified solver package and also checks if all other arguments (`method` and `pType`) are valid arguments. Each solver package has its own set of methods for specific types of optimization (e. g. linear programming or quadratic programming) and is thus available maybe not for all problem types.

Initialize the new problem object. Each solver software needs to create specific data structures to hold the problem and solution data.

```
> lp <- initProb(lp)
```

```
linear programming problem object
solver: glpkAPI
method: exact
problem is currently empty
```

Slot `oobj` holds a pointer to the problem object of GLPK. Now, we need to allocate space for the problem data and load the data into the problem object.

```
> cm <- Matrix(c(0.5, 2, 1, 1), nrow = 2)
> loadLPprob(lp, nCols = 2, nRows = 2, mat = cm,
+            lb = c(0, 0), ub = rep(1000, 2), obj = c(1, 1),
+            rlb = c(0, 0), rub = c(4.5, 9), rtype = c("U", "U"),
+            lpdir = "max")
```

The first command generates the constraint matrix in sparse format (see also documentation in package *Matrix*). The second command loads the problem data into the problem object.

```
> lp

linear programming problem object
solver: glpkAPI
method: exact
problem has 2 variables and 2 constraints
```

All data are now set in the problem object, so it can be solved.

```
> status <- solveLp(lp)

[1] 0
```

Translate the status code in a text string.

```
> getMeanReturn(code = status, solver = solver(lp))

[1] "solution process was successful"
```

Check the solution status.

```
> status <- getSolStat(lp)
> getMeanStatus(code = status, solver = solver(lp))

[1] "solution is optimal"
```

Retrieve the value of the objective function and the values of the variables after optimization.

```
> getObjVal(lp)

[1] 6

> getFluxDist(lp)

[1] 3 3
```

Get the reduced costs.

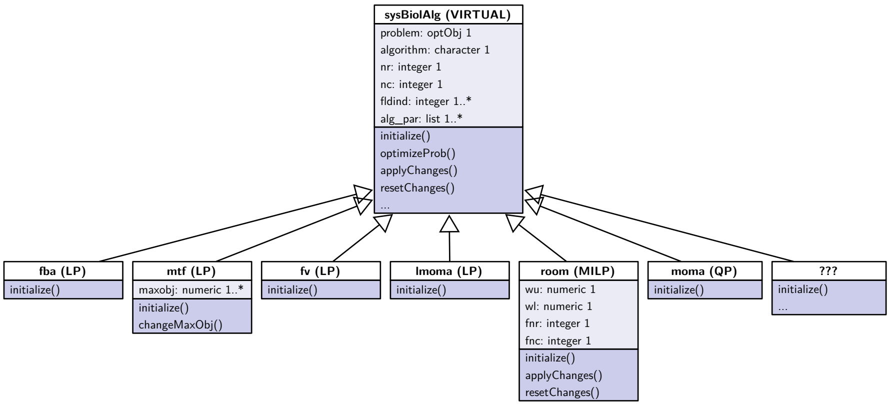

Figure 5: UML representation of class `sysBiolAlg`.

```
> getRedCosts(lp)

[1] 0 0
```

Delete problem object and free all memory allocated by the solver software.

```
> delProb(lp)
> lp

linear programming problem object
solver: glpkAPI
method: exact
problem is not initialized
```

5.4 Class sysBiolAlg

The class `sysBiolAlg` holds objects of class `optObj` which are prepared for a particular kind of algorithm, e. g. FBA, MOMA or ROOM (figure 5). Class `optObj` takes care of the communication between *sybil* and the solver software. Class `sysBiolAlg` instead is responsible for the algorithm used to analyze a metabolic network. The constructor function `sysBiolalg()` (figure 6) gets at least two arguments: 1. an object of class `modelorg` (section 5.1) and 2. a single character string indicating the name of the desired algorithm. Further arguments are passed through argument `...` to the corresponding constructor of the class extending class `sysBiolAlg`. The base class `sysBiolAlg` is virtual, no objects can be created from that class directly. The constructor function builds an instance of a class extending the base class:

```
> ec <- sysBiolAlg(Ec_core, algorithm = "fba")
> is(ec)

[1] "sysBiolAlg_fba" "sysBiolAlg"
```

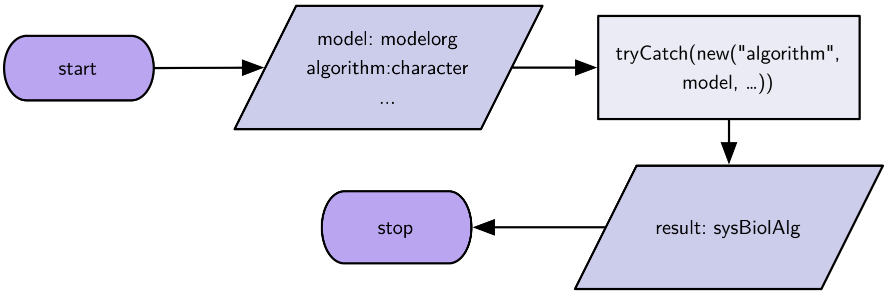

Figure 6: Work flow of the constructor function `sysBiolAlg()`.

Now, the variable `ec` contains an object of class `sysBiolAlg_fba`. Slot `problem` of that object is of class `optObj` and is prepared for FBA. The optimization can be performed with method `optimizeProb`:

```
> opt <- optimizeProb(ec)
```

The return value of `optimizeProb` is discussed in section 4.4. Method `optimizeProb` of class `sysBiolAlg` always returns a list, not an object of class `optsol`. In order to run a ROOM analysis create an object of class `sysBiolAlg_room`:

```
> ecr <- sysBiolAlg(Ec_core, algorithm = "room", wtflux = opt$fluxes)
> is(ecr)

[1] "sysBiolAlg_room" "sysBiolAlg"

> ecr

Algorithm type: room
Slot problem:
mixed integer linear programming problem object
solver: glpkAPI
method: mip
problem has 190 variables and 262 constraints
Slot fldind:
 int [1:95] 1 2 3 4 5 6 7 8 9 10 ...
```

Argument `wtflux` gets the optimal flux distribution computed via FBA earlier. It is used by the constructor method of class `sysBiolAlg_room`.

5.4.1 Constructor methods

The base class `sysBiolAlg` contains a constructor method `initialize` which is called by the constructor methods of the subclasses via `callNextMethod()` (figure 7). Every subclass has its own constructor method preparing all necessary data structures in order to

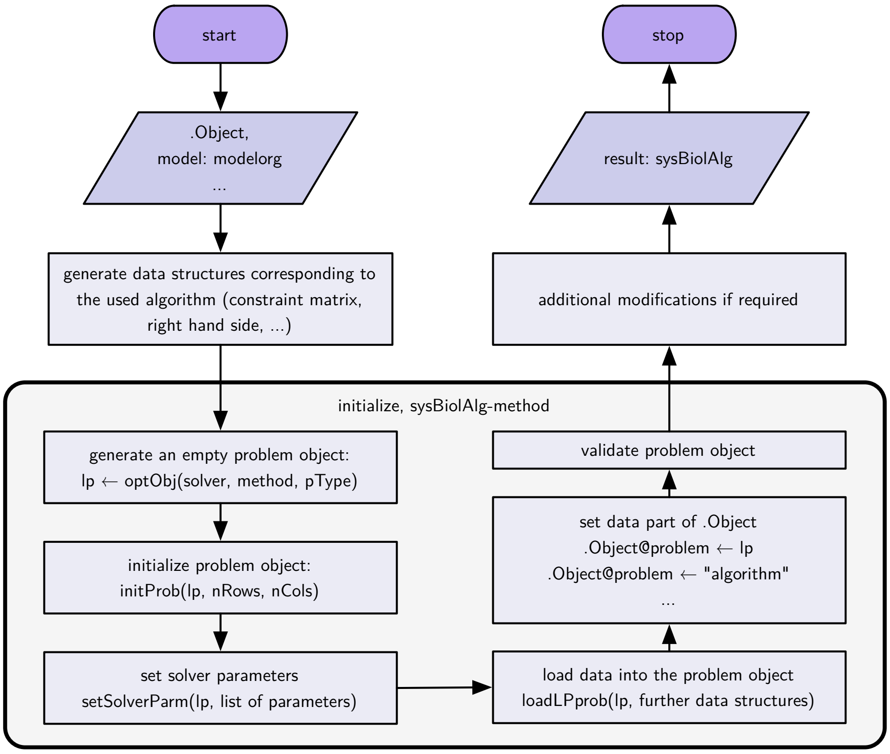

Figure 7: Work flow of the constructor methods of classes extending class `sysBiolAlg`. The gray shaded part is done by the constructor method or the base class.

call the constructor of the base class. For example, for the ROOM algorithm, a "wild type" flux distribution is required (argument `wtflux` in the example above). The constructor of class `sysBiolAlg_room` generates all data structures to build the optimization problem, e. g. the constraint matrix, objective coefficients, right hand side, . . . It passes all these data to the constructor of `sysBiolAlg` via a call to `callNextMethod()`. This constructor generates the object of class `optObj` while taking care on solver software specific details.

5.4.2 New algorithms

In order to extend the functionality of *sybil* with new algorithms, a new class describing that algorithm is required. The function `promptSysBiolAlg()` generates a skeletal structure of a new class definition and a corresponding constructor method. To implement an algorithm named "foo", run

```
> promptSysBiolAlg(algorithm = "foo")
```

which generates a file `sysBiolAlg_fooClass.R` containing the new class definition. The class `sysBiolAlg_foo` will extend class `sysBiolAlg` directly and will not add any slots to the class. Additionally, an unfinished method `initialize` is included. Here it is

necessary to generate the data structures required by the new algorithm. There are comments in the skeletal structure guiding through the process.

References

S. A. Becker et al. Quantitative prediction of cellular metabolism with constraint-based models: the COBRA Toolbox. *Nat Protoc*, 2(3):727–738, 2007. doi: 10.1038/nprot.2007.99.

J. S. Edwards, R. U. Ibarra, and B. Ø. Palsson. In silico predictions of *Escherichia coli* metabolic capabilities are consistent with experimental data. *Nat Biotechnol*, 19(2): 125–130, Feb 2001. doi: 10.1038/84379.

J. S. Edwards, M. Covert, and B. Ø. Palsson. Metabolic modelling of microbes: the flux-balance approach. *Environ Microbiol*, 4(3):133–140, Mar 2002a.

J. S. Edwards, R. Ramakrishna, and B. Ø. Palsson. Characterizing the metabolic phenotype: a phenotype phase plane analysis. *Biotechnol Bioeng*, 77(1):27–36, Jan 2002b.

R. Mahadevan and C. H. Schilling. The effects of alternate optimal solutions in constraint-based genome-scale metabolic models. *Metab Eng*, 5(4):264–276, Oct 2003.

J. D. Orth, R. M. T. Fleming, and B. Ø. Palsson. Reconstruction and use of microbial metabolic networks: the core *Escherichia coli* metabolic model as an educational guide. EcoSal Chapter 10.2.1, 2010a.

J. D. Orth, I. Thiele, and B. Ø. Palsson. What is flux balance analysis? *Nat Biotechnol*, 28(3):245–248, Mar 2010b. doi: 10.1038/nbt.1614.

B. Ø. Palsson. *Systems Biology: Properties of Recontructed Networks*. Cambridge University Press, 2006.

J. Schellenberger, J. O. Park, T. M. Conrad, and B. Ø. Palsson. BiGG: a biochemical genetic and genomic knowledgebase of large scale metabolic reconstructions. *BMC Bioinformatics*, 11:213, 2010. doi: 10.1186/1471-2105-11-213.

J. Schellenberger, R. Que, R. M. T. Fleming, I. Thiele, J. D. Orth, A. M. Feist, D. C. Zielinski, A. Bordbar, N. E. Lewis, S. Rahmanian, J. Kang, D. R. Hyduke, and B. Ø. Palsson. Quantitative prediction of cellular metabolism with constraint-based models: the COBRA Toolbox v2.0. *Nat Protoc*, 6(9):1290–1307, 2011. doi: 10.1038/nprot.2011.308.

D. Segrè et al. Analysis of optimality in natural and perturbed metabolic networks. *Proc Natl Acad Sci U S A*, 99(23):15112–15117, Nov 2002. doi: 10.1073/pnas.232349399.

T. Shlomi, O. Berkman, and E. Ruppin. Regulatory on/off minimization of metabolic flux changes after genetic perturbations. *Proc Natl Acad Sci U S A*, 102(21):7695–7700, May 2005. doi: 10.1073/pnas.0406346102.
