# Supplementary material for: sybil – Efficient constraint-based modelling in R
Source: BMC Syst Biol. 2013 Nov 13;7:125. doi: 10.1186/1752-0509-7-125 (PMC3843580; doi:10.1186/1752-0509-7-125)
Supplement: Additional file 1 — R package sybil. Archive of the sybil version current at submission. [file 1752-0509-7-125-S1.zip › sybil_1.2.4.tar.gz!/sybil/vignettes/net-crop.pdf]

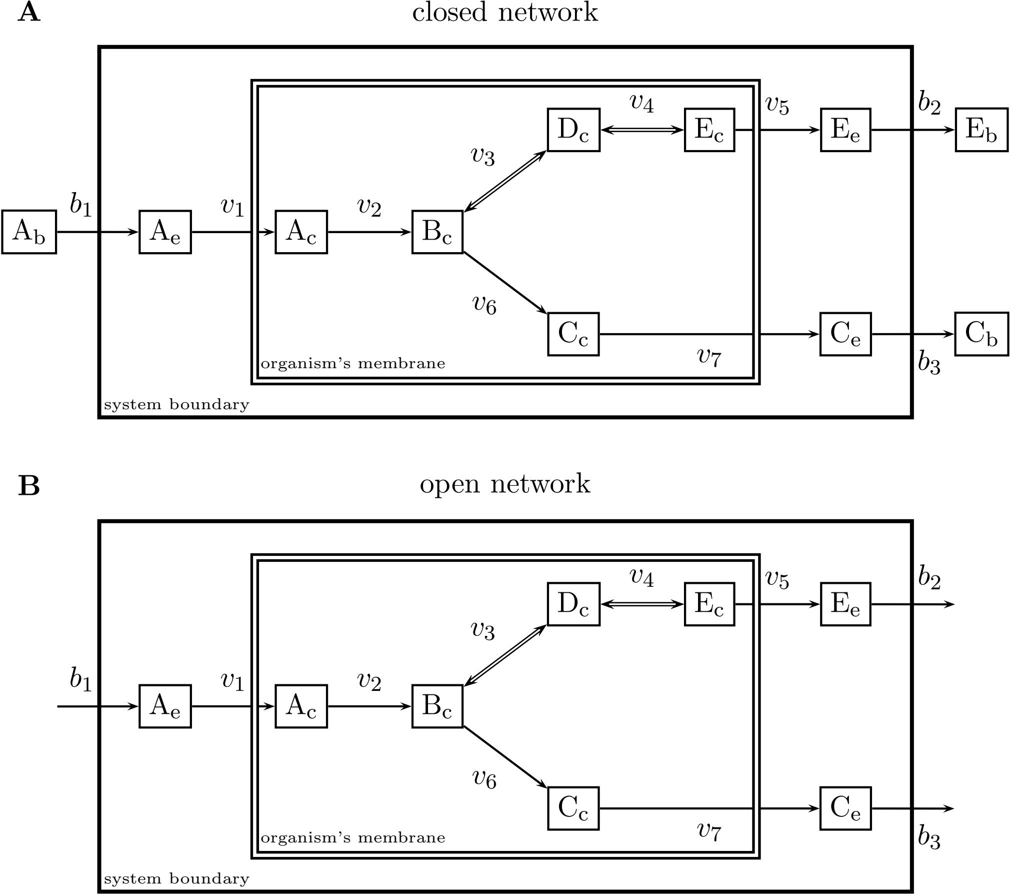
A
closed network
system boundary
organism's membrane
A_b
b_1
A_e
v_1
A_c
v_2
B_c
v_3
D_c
v_4
E_c
v_5
E_e
b_2
E_b
v_6
C_c
v_7
C_e
b_3
C_b
B
open network
system boundary
organism's membrane
b_1
A_e
v_1
A_c
v_2
B_c
v_3
D_c
v_4
E_c
v_5
E_e
b_2
v_6
C_c
v_7
C_e
b_3
