# Supplementary material for: sybil – Efficient constraint-based modelling in R
Source: BMC Syst Biol. 2013 Nov 13;7:125. doi: 10.1186/1752-0509-7-125 (PMC3843580; doi:10.1186/1752-0509-7-125)
Supplement: Additional file 1 — R package sybil. Archive of the sybil version current at submission. [file 1752-0509-7-125-S1.zip › sybil_1.2.4.tar.gz!/sybil/vignettes/optObj-class.pdf]

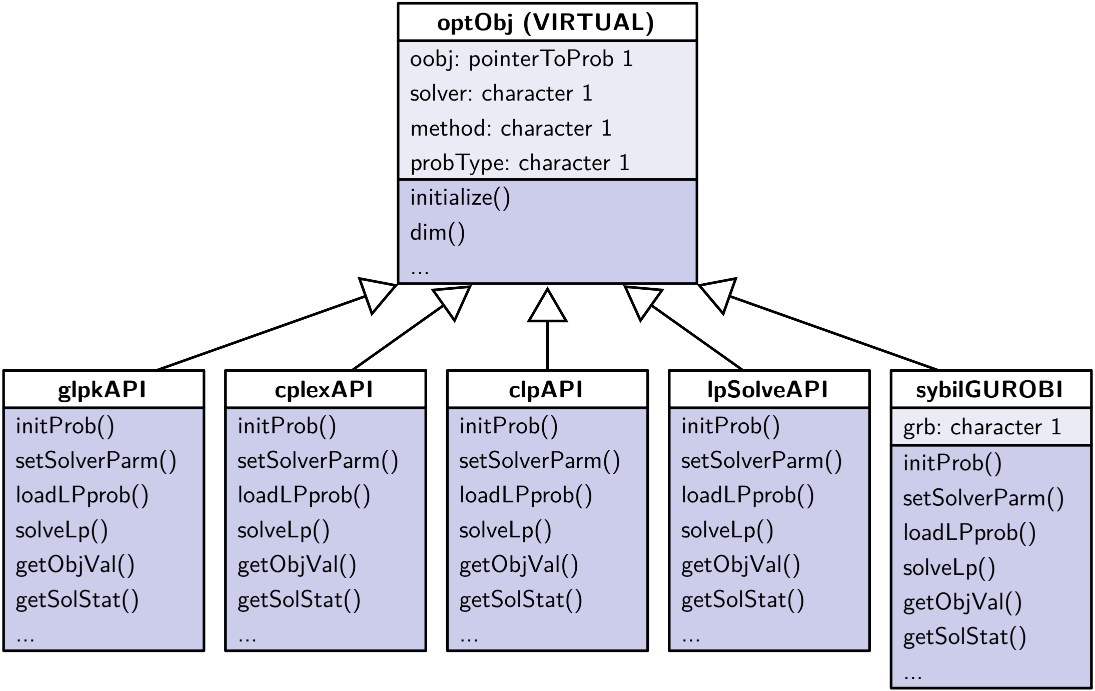
optObj (VIRTUAL)
oobj: pointerToProb 1
solver: character 1
method: character 1
probType: character 1
initialize()
dim()
...
glpkAPI
initProb()
setSolverParm()
loadLPprob()
solveLp()
getObjVal()
getSolStat()
...
cplexAPI
initProb()
setSolverParm()
loadLPprob()
solveLp()
getObjVal()
getSolStat()
...
clpAPI
initProb()
setSolverParm()
loadLPprob()
solveLp()
getObjVal()
getSolStat()
...
lpSolveAPI
initProb()
setSolverParm()
loadLPprob()
solveLp()
getObjVal()
getSolStat()
...
sybilGUROBI
grb: character 1
initProb()
setSolverParm()
loadLPprob()
solveLp()
getObjVal()
getSolStat()
...
