# Supplementary material for: sybil – Efficient constraint-based modelling in R
Source: BMC Syst Biol. 2013 Nov 13;7:125. doi: 10.1186/1752-0509-7-125 (PMC3843580; doi:10.1186/1752-0509-7-125)
Supplement: Additional file 1 — R package sybil. Archive of the sybil version current at submission. [file 1752-0509-7-125-S1.zip › sybil_1.2.4.tar.gz!/sybil/vignettes/optsol-class.pdf]

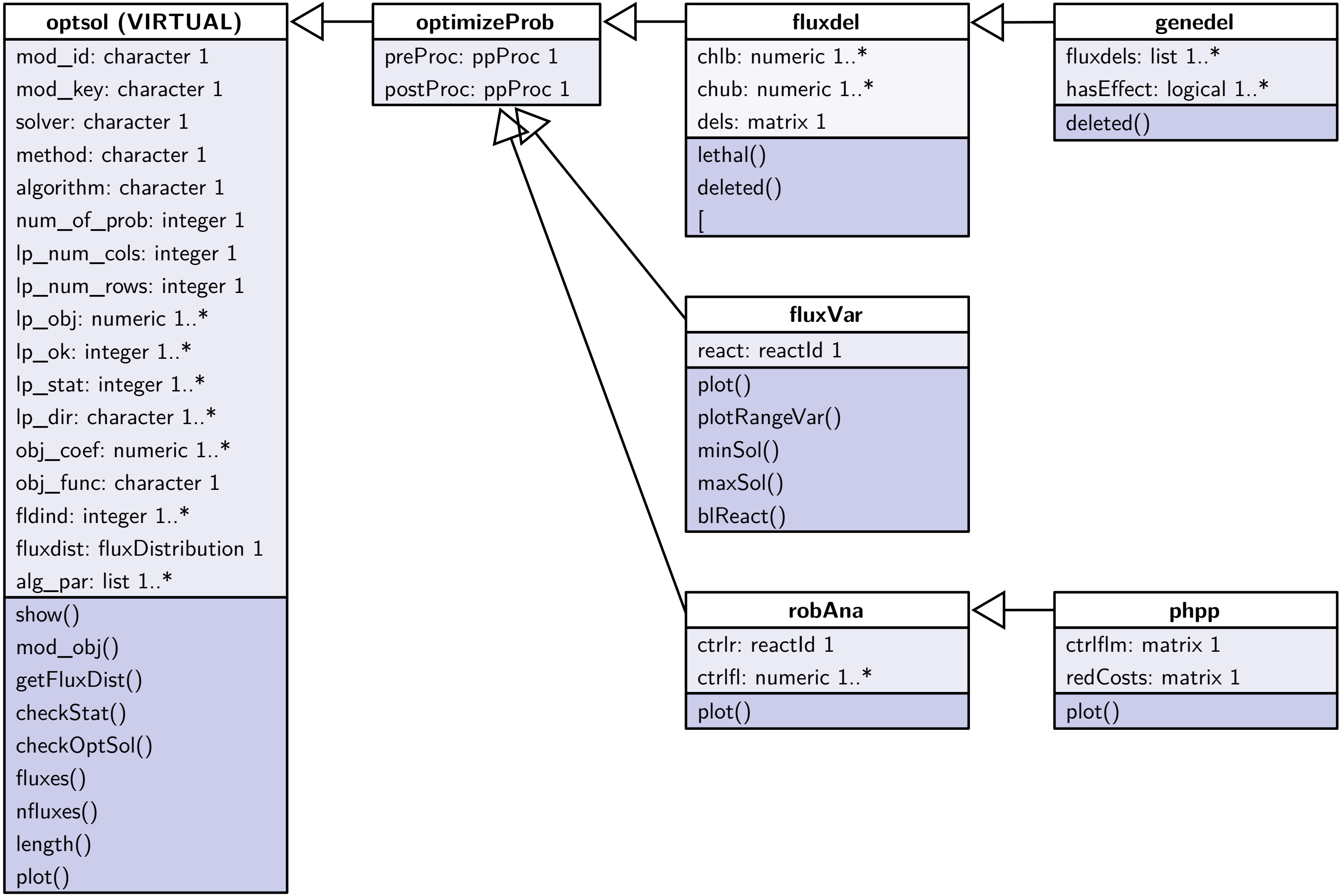
optsol (VIRTUAL)
mod_id: character 1
mod_key: character 1
solver: character 1
method: character 1
algorithm: character 1
num_of_prob: integer 1
lp_num_cols: integer 1
lp_num_rows: integer 1
lp_obj: numeric 1..*
lp_ok: integer 1..*
lp_stat: integer 1..*
lp_dir: character 1..*
obj_coef: numeric 1..*
obj_func: character 1
fldind: integer 1..*
fluxdist: fluxDistribution 1
alg_par: list 1..*
show()
mod_obj()
getFluxDist()
checkStat()
checkOptSol()
fluxes()
nfluxes()
length()
plot()
optimizeProb
preProc: ppProc 1
postProc: ppProc 1
fluxdel
chlb: numeric 1..*
chub: numeric 1..*
dels: matrix 1
lethal()
deleted()
[
genedel
fluxdels: list 1..*
hasEffect: logical 1..*
deleted()
fluxVar
react: reactId 1
plot()
plotRangeVar()
minSol()
maxSol()
blReact()
robAna
ctrlr: reactId 1
ctrlfl: numeric 1..*
plot()
phpp
ctrlflm: matrix 1
redCosts: matrix 1
plot()
