# Supplementary material for: sybil – Efficient constraint-based modelling in R
Source: BMC Syst Biol. 2013 Nov 13;7:125. doi: 10.1186/1752-0509-7-125 (PMC3843580; doi:10.1186/1752-0509-7-125)
Supplement: Additional file 1 — R package sybil. Archive of the sybil version current at submission. [file 1752-0509-7-125-S1.zip › sybil_1.2.4.tar.gz!/sybil/vignettes/sysBiolAlg-class.pdf]

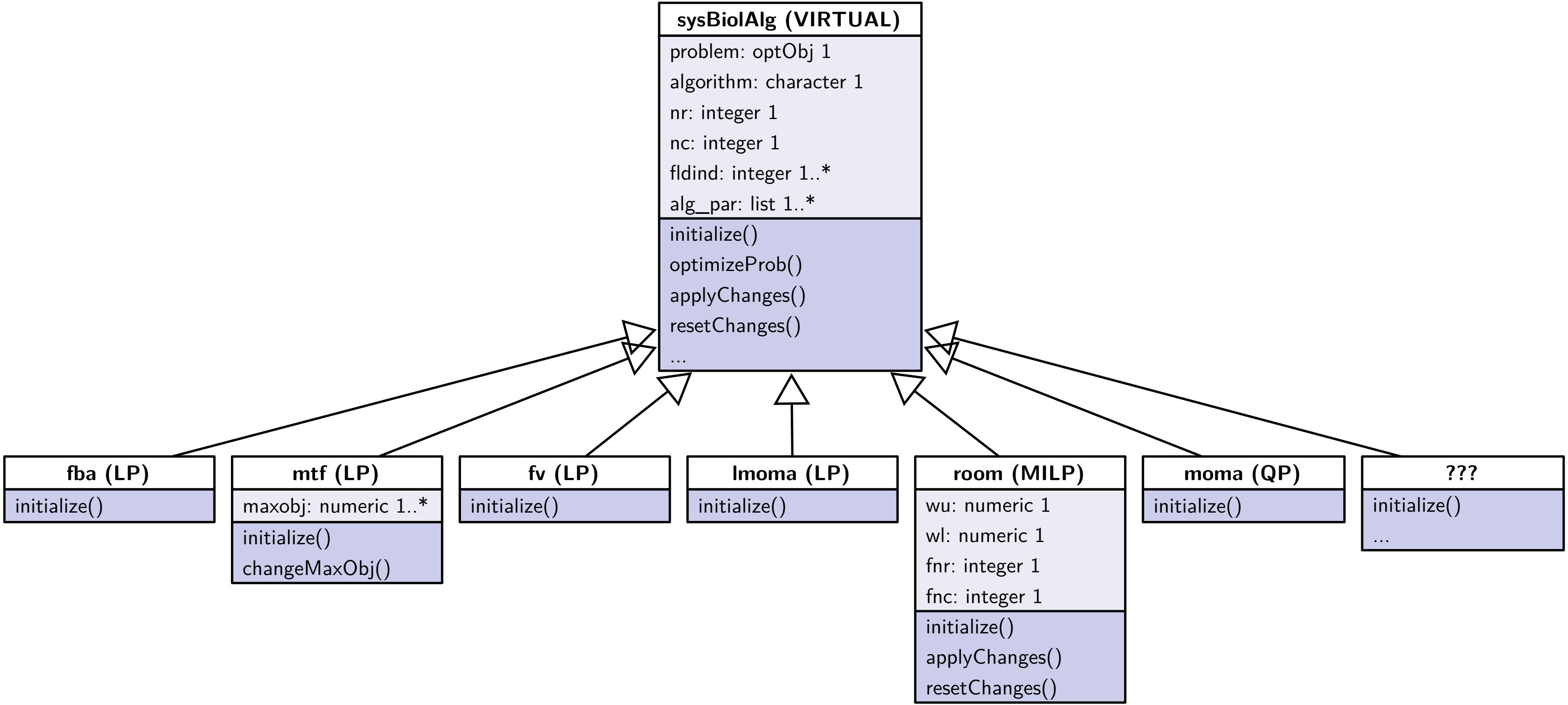
sysBiolAlg (VIRTUAL)
problem: optObj 1
algorithm: character 1
nr: integer 1
nc: integer 1
fldind: integer 1..*
alg_par: list 1..*
initialize()
optimizeProb()
applyChanges()
resetChanges()
...
fba (LP)
initialize()
mtf (LP)
maxobj: numeric 1..*
initialize()
changeMaxObj()
fv (LP)
initialize()
lmoma (LP)
initialize()
room (MILP)
wu: numeric 1
wl: numeric 1
fnr: integer 1
fnc: integer 1
initialize()
applyChanges()
resetChanges()
moma (QP)
initialize()
???
initialize()
...
