# Supplementary material for: sybil – Efficient constraint-based modelling in R
Source: BMC Syst Biol. 2013 Nov 13;7:125. doi: 10.1186/1752-0509-7-125 (PMC3843580; doi:10.1186/1752-0509-7-125)
Supplement: Additional file 1 — R package sybil. Archive of the sybil version current at submission. [file 1752-0509-7-125-S1.zip › sybil_1.2.4.tar.gz!/sybil/vignettes/sysBiolAlg-const.pdf]

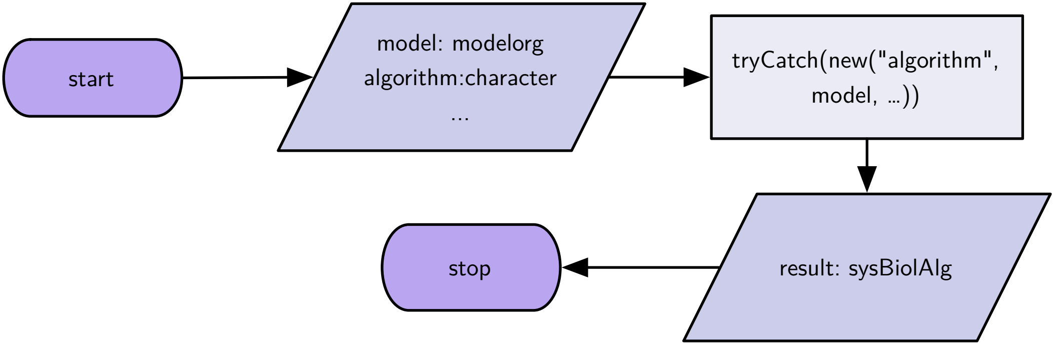
start
model: modelorg
algorithm:character
...
tryCatch(new("algorithm",
model, ...))
result: sysBiolAlg
stop
