# Supplementary material for: sybil – Efficient constraint-based modelling in R
Source: BMC Syst Biol. 2013 Nov 13;7:125. doi: 10.1186/1752-0509-7-125 (PMC3843580; doi:10.1186/1752-0509-7-125)
Supplement: Additional file 1 — R package sybil. Archive of the sybil version current at submission. [file 1752-0509-7-125-S1.zip › sybil_1.2.4.tar.gz!/sybil/vignettes/sysBiolAlg-init.pdf]

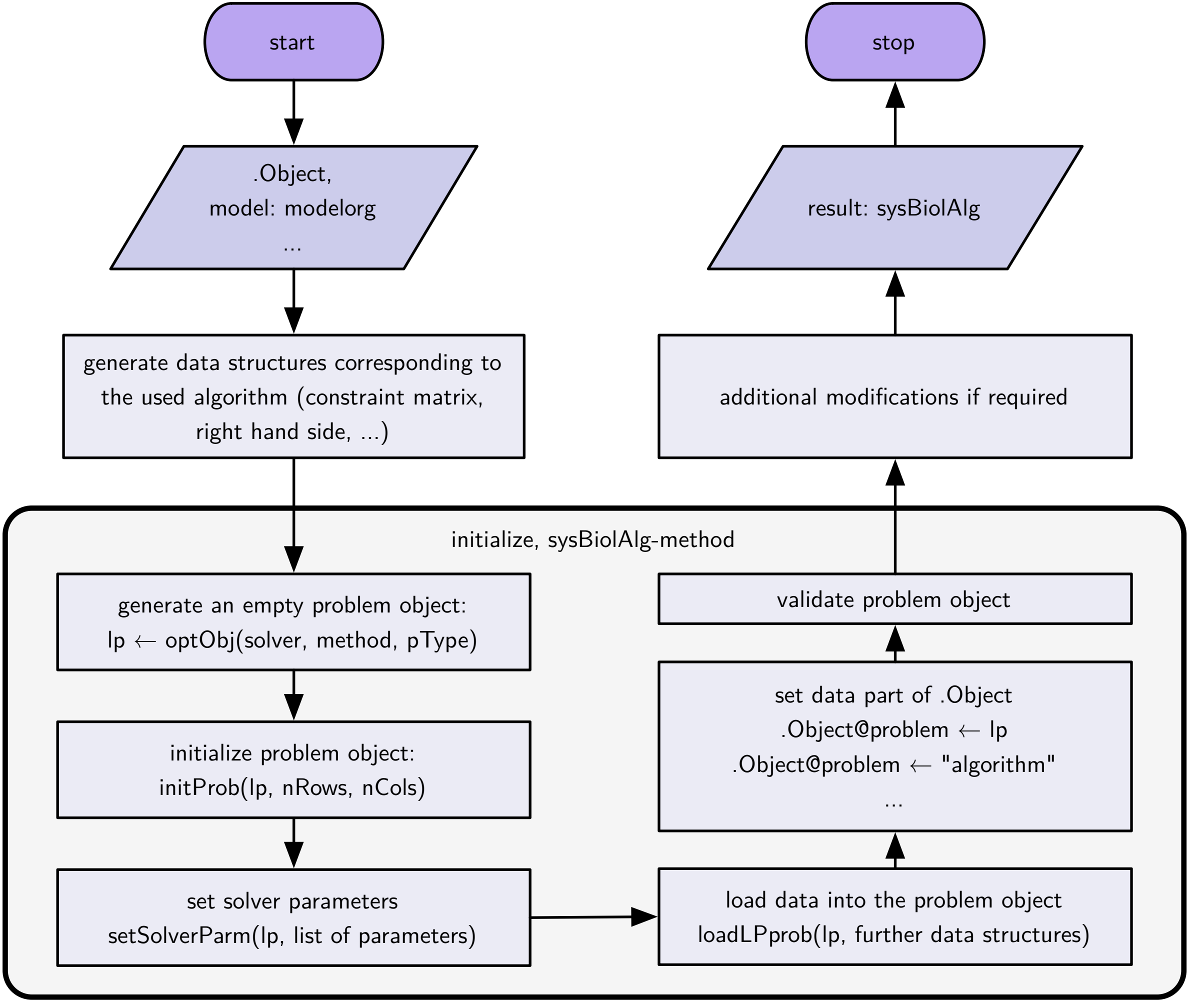
start
.Object,
model: modelorg
...
generate data structures corresponding to the used algorithm (constraint matrix, right hand side, ...)
initialize, sysBiolAlg-method
generate an empty problem object:
lp ← optObj(solver, method, pType)
initialize problem object:
initProb(lp, nRows, nCols)
set solver parameters
setSolverParm(lp, list of parameters)
load data into the problem object
loadLPprob(lp, further data structures)
set data part of .Object
.Object@problem ← lp
.Object@problem ← "algorithm"
...
validate problem object
additional modifications if required
result: sysBiolAlg
stop
