# Supplementary material for: sybil – Efficient constraint-based modelling in R
Source: BMC Syst Biol. 2013 Nov 13;7:125. doi: 10.1186/1752-0509-7-125 (PMC3843580; doi:10.1186/1752-0509-7-125)
Supplement: Additional file 3 — R package sybilSBML. Archive of the sybilSBML version current at submission. [file 1752-0509-7-125-S3.zip › sybilSBML_2.0.6.tar.gz!/sybilSBML/inst/doc/sybilSBML.pdf]

sybilSBML – Quick Start

Gabriel Gelius-Dietrich

October 26, 2013

1 Introduction

The package *sybilSBML* is an addition to package *sybil*[1] providing support for metabolic networks written in SBML (Systems Biology Markup Language), in particular those developed by Bernhard Ø. Palsson's lab[2] and those from the BiGG database[3] [Schellenberger et al., 2010].

2 Installation

The package *sybilSBML* depends on a working installation of LibSBML [Bornstein et al., 2008] available from the SBML homepage[4] (in particular libraries and header files). See `INSTALL` for installation instructions and platform specific details.

3 Usage

The package *sybilSBML* provides the command `readSBMLmod()` which reads SBML formated files and returns instances of class `modelorg`.

```
> library(sybilSBML)
> model <- readSBMLmod("<model>.xml")
```

4 Input files

The function `readSBMLmod()` reads metabolic network models written in SBML format (Systems Biology Markup Language). Among the models available in this de-facto standard format are in particular those developed by Bernhard Ø. Palsson's lab.

The file `ecoli_core_model.xml` (in `extdata/`) contains an exemplarily metabolic network written in SBML for the core energy metabolism of *E. coli* [Palsson, 2006, Orth

[1] `http://CRAN.R-project.org/package=sybil`
[2] `http://gcrg.ucsd.edu/`
[3] `http://bigg.ucsd.edu/`
[4] `http://www.sbml.org/`, libSBML version 5.6.0 or higher

et al., 2010]. The exact location of the file can be retrieved with the `system.file()` command:

```
> library(sybilSBML)
> mp     <- system.file(package = "sybilSBML", "extdata")
> ec_mod <- file.path(mp, "ecoli_core_model.xml")
```

The model can be read in by using the command `readSBMLmod()`:

```
> mod <- readSBMLmod(ec_mod, bndCond = FALSE)
```

```
model name:              Ecoli_core_model
number of compartments   2
                         C_c
                         C_e
number of reactions:     95
number of metabolites:   72
number of unique genes:  137
objective function:      +1 Biomass_Ecoli_core_w_GAM
```

The metabolite id's of the SBML files are written in the format `M_<metabolite abbreviation>_<compartment abbreviation>`. The compartment abbreviation is a one letter abbreviation, e. g. `c` for cytosol. All metabolites outside the system boundary belong to compartment `b`. Those metabolites are transported into or outside the system. As long as they are mentioned, the network is closed. The function `readSBMLmod()` will remove them in order to produce an open network.

5 Validation of input files

SBML files can be validated by using the command `validateSBMLdocument()`:

```
> err <- validateSBMLdocument(ec_mod)
```

The variable `err` is of class `sbmlError`, storing error messages generated by the validation procedure.

References

S. A. Becker et al. Quantitative prediction of cellular metabolism with constraint-based models: the COBRA Toolbox. *Nat Protoc*, 2(3):727–738, 2007. doi: 10.1038/nprot.2007.99.

B. J. Bornstein et al. LibSBML: an API library for SBML. *Bioinformatics*, 24(6):880–881, Mar 2008. doi: 10.1093/bioinformatics/btn051.

J. D. Orth, R. M. T. Fleming, and B. Ø. Palsson. Reconstruction and use of microbial metabolic networks: the core *Escherichia coli* metabolic model as an educational guide. EcoSal Chapter 10.2.1, 2010.

B. Ø. Palsson. *Systems Biology: Properties of Recontructed Networks*. Cambridge University Press, 2006.

J. Schellenberger, J. O. Park, T. M. Conrad, and B. Ø. Palsson. BiGG: a biochemical genetic and genomic knowledgebase of large scale metabolic reconstructions. *BMC Bioinformatics*, 11:213, 2010. doi: 10.1186/1471-2105-11-213.

J. Schellenberger, R. Que, R. M. T. Fleming, I. Thiele, J. D. Orth, A. M. Feist, D. C. Zielinski, A. Bordbar, N. E. Lewis, S. Rahmanian, J. Kang, D. R. Hyduke, and B. Ø. Palsson. Quantitative prediction of cellular metabolism with constraint-based models: the COBRA Toolbox v2.0. *Nat Protoc*, 6(9):1290–1307, 2011. doi: 10.1038/nprot.2011.308.
